# Supplementary figures and images for: Salmonella-induced SIRT1 and SIRT3 are crucial for maintaining the metabolic switch in bacteria and host for successful pathogenesis
Source: eLife. 2024 Dec 18;13:RP93125. doi: 10.7554/eLife.93125 (PMC11655064; doi:10.7554/eLife.93125)

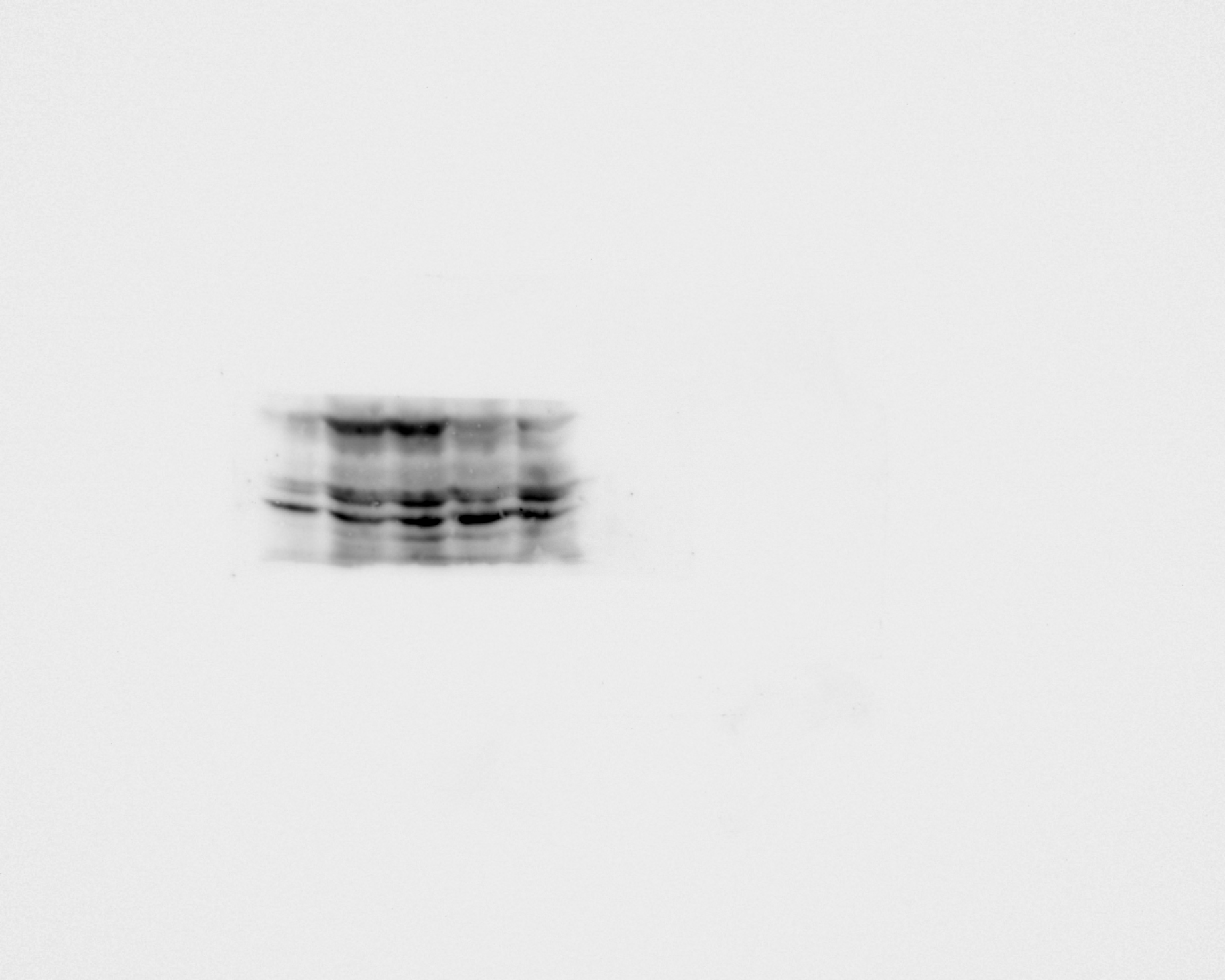

Supplement: Figure 1—figure supplement 1—source data 2. [file elife-93125-fig1-figsupp1-data2.zip › DH DC Lab 2024-04-10 18h26m12s(Chemiluminescence).tif]

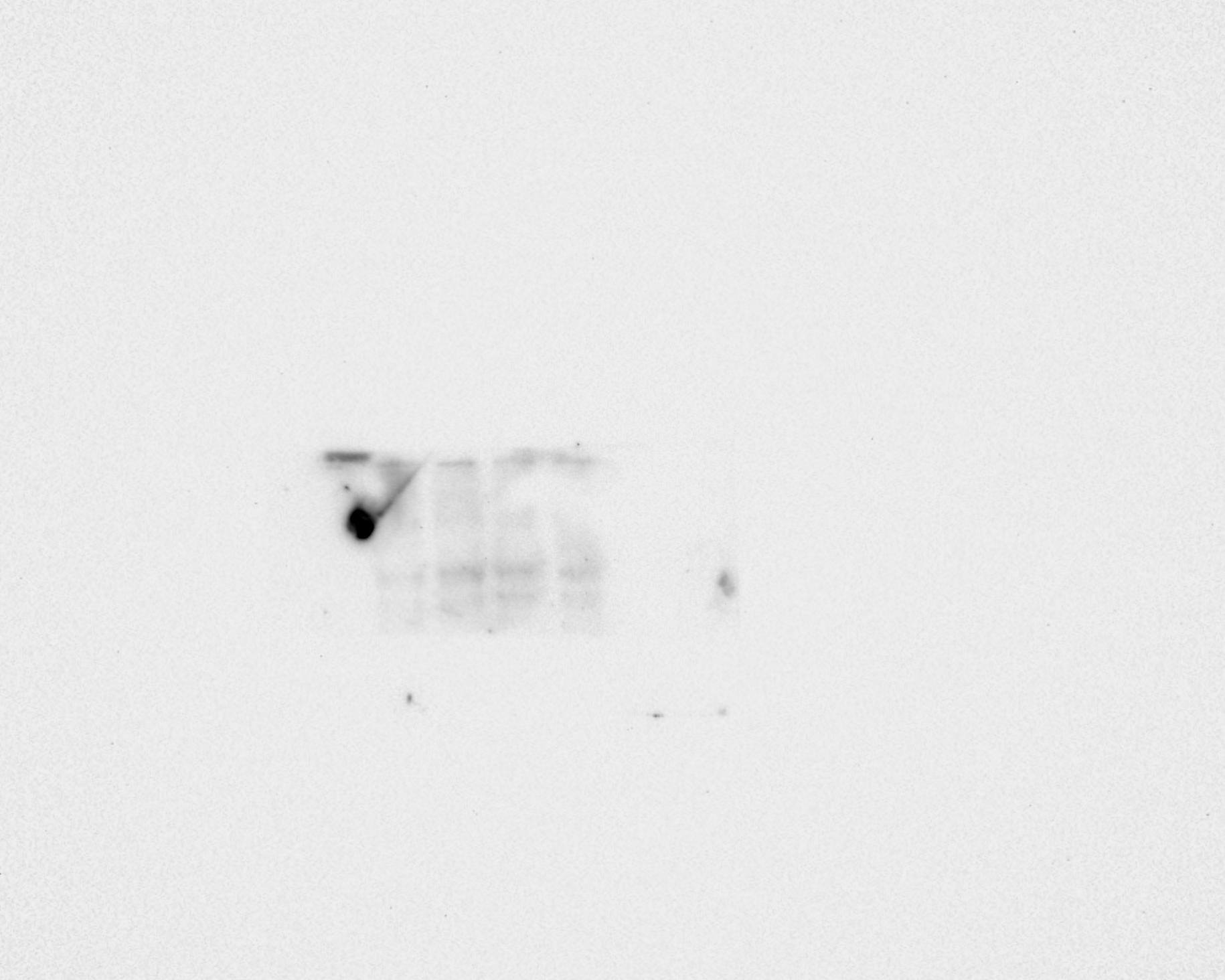

Supplement: Figure 1—figure supplement 1—source data 2. [file elife-93125-fig1-figsupp1-data2.zip › DH DC Lab 2024-04-10 18h11m30s(Chemiluminescence).tif]

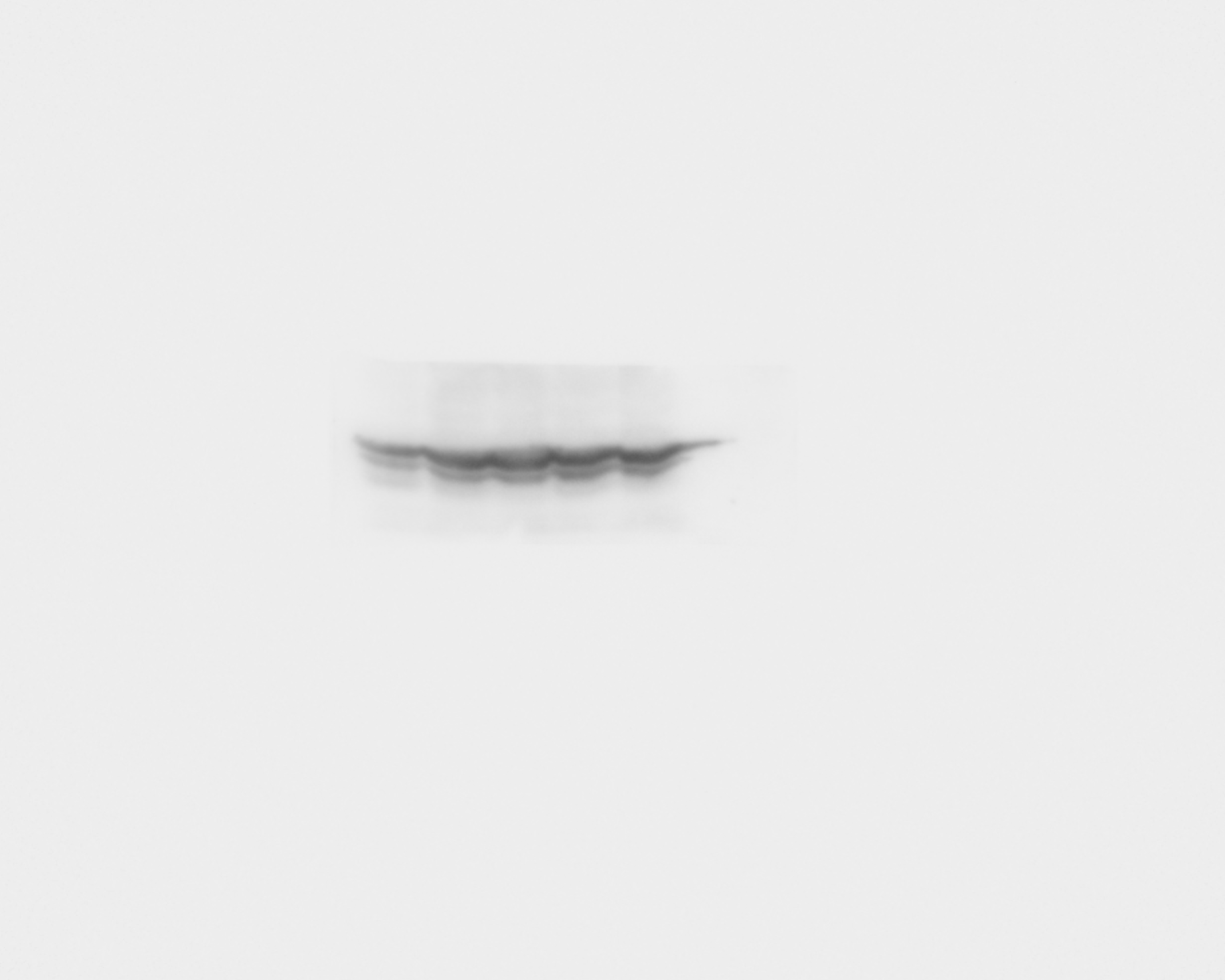

Supplement: Figure 1—figure supplement 1—source data 2. [file elife-93125-fig1-figsupp1-data2.zip › S1 B b actin.tif]

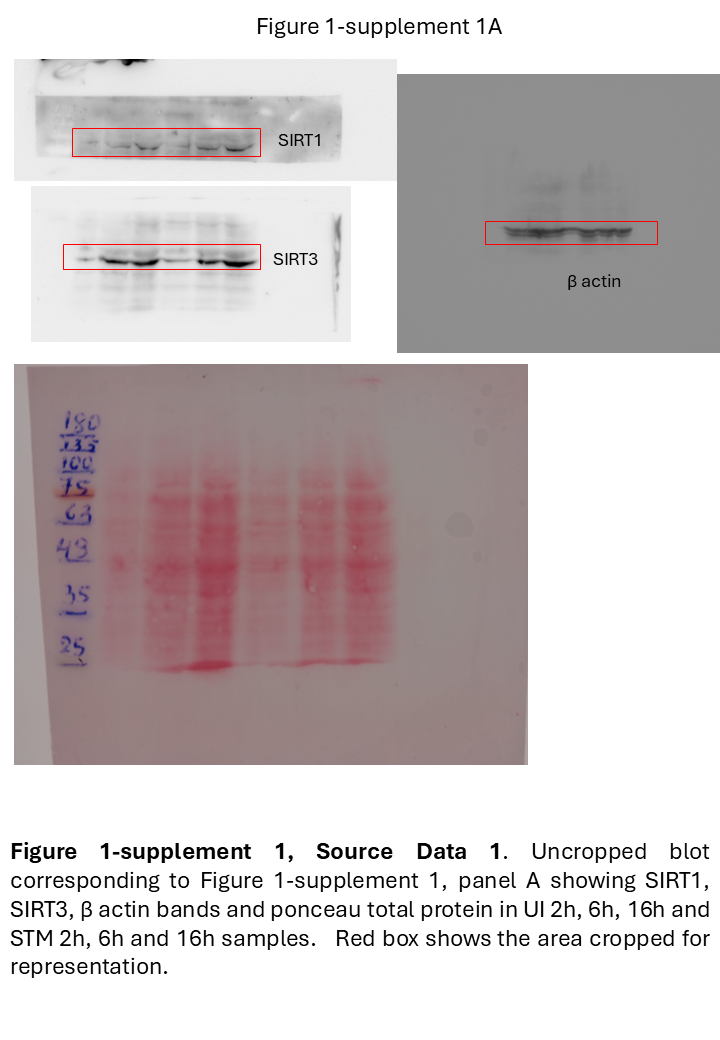

Supplement: Figure 1—figure supplement 1—source data 3. [file elife-93125-fig1-figsupp1-data3.zip › Figure 1-supplement 1-Source Data 1.TIF]

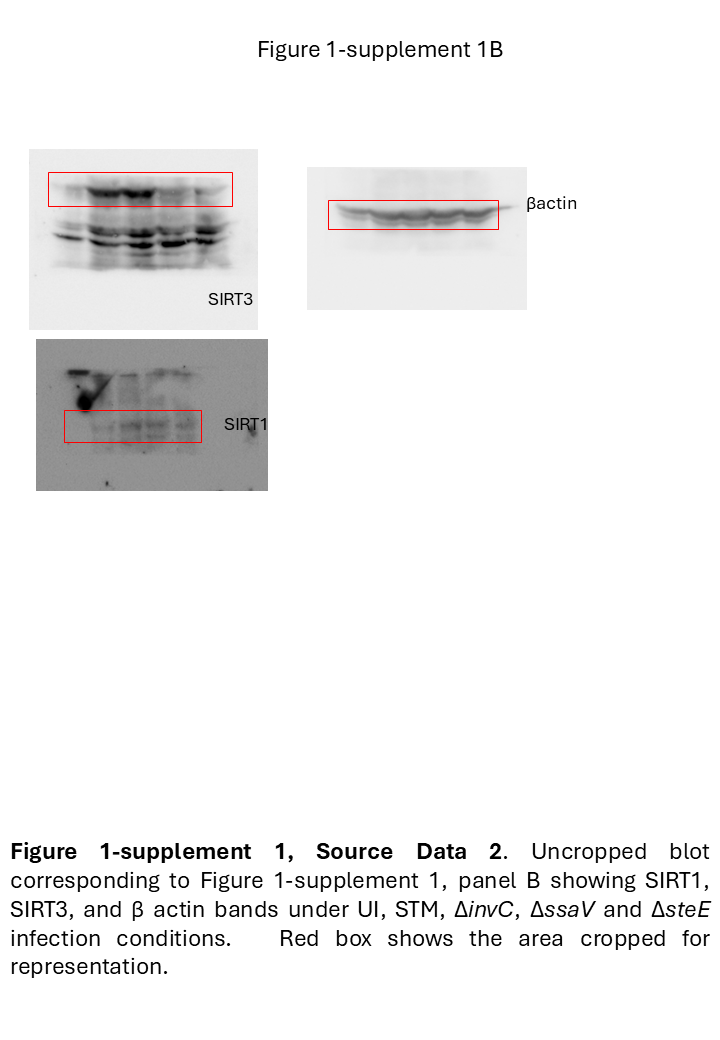

Supplement: Figure 1—figure supplement 1—source data 4. [file elife-93125-fig1-figsupp1-data4.zip › Figure 1-supplement 1-Source Data 2.TIF]

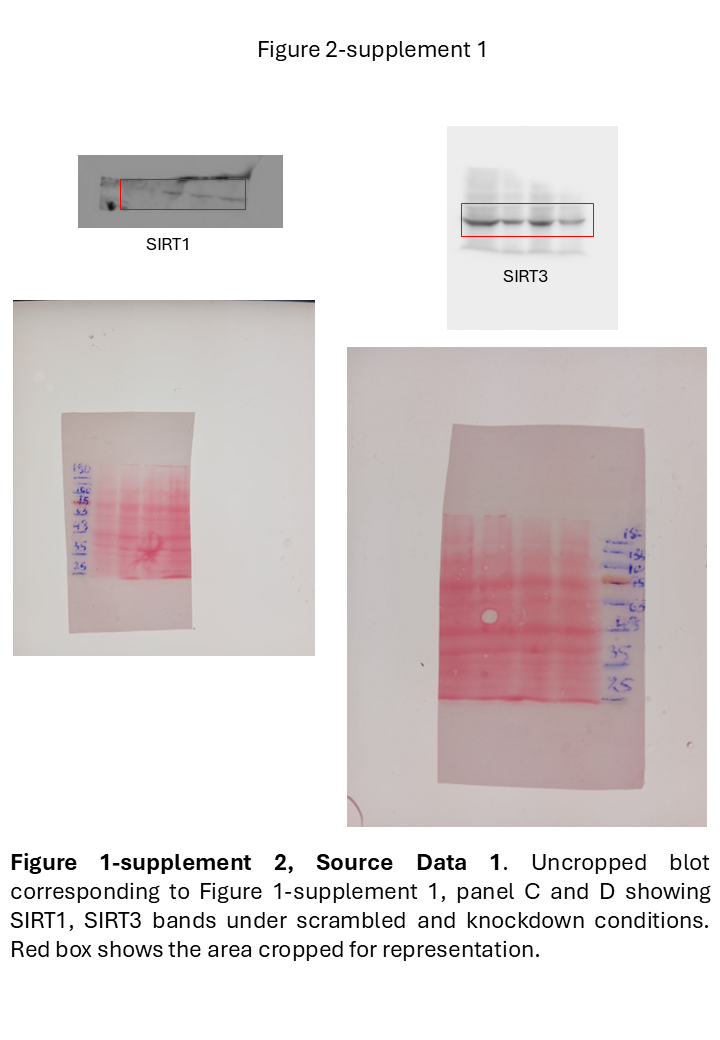

Supplement: Figure 2—figure supplement 1—source data 2. [file elife-93125-fig2-figsupp1-data2.zip › Figure 2-supplement 1-Source Data 1.TIF]

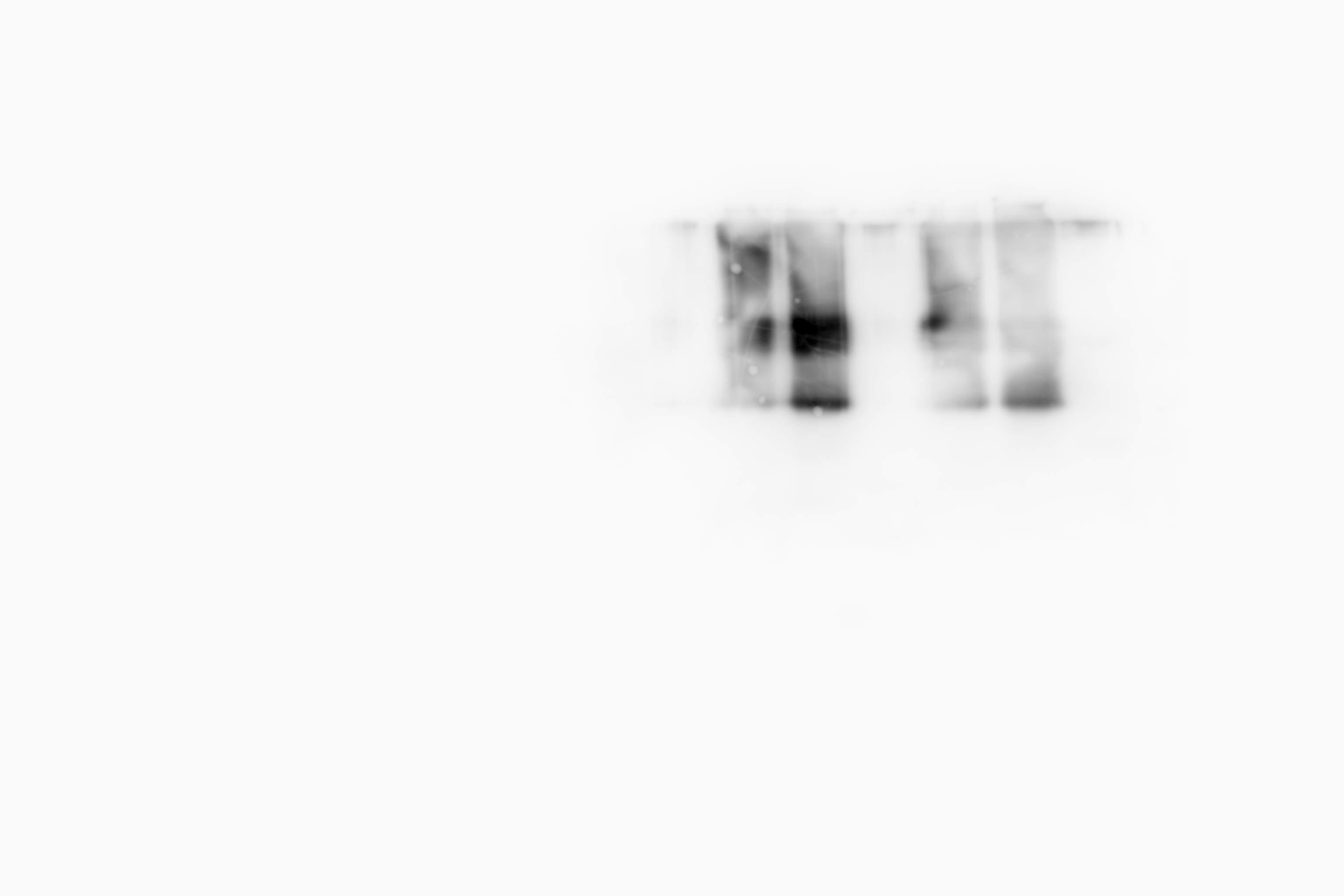

Supplement: Figure 4—source data 1. [file elife-93125-fig4-data1.zip › IP- SIRT1 in control IP p65 UI STM_4.tif]

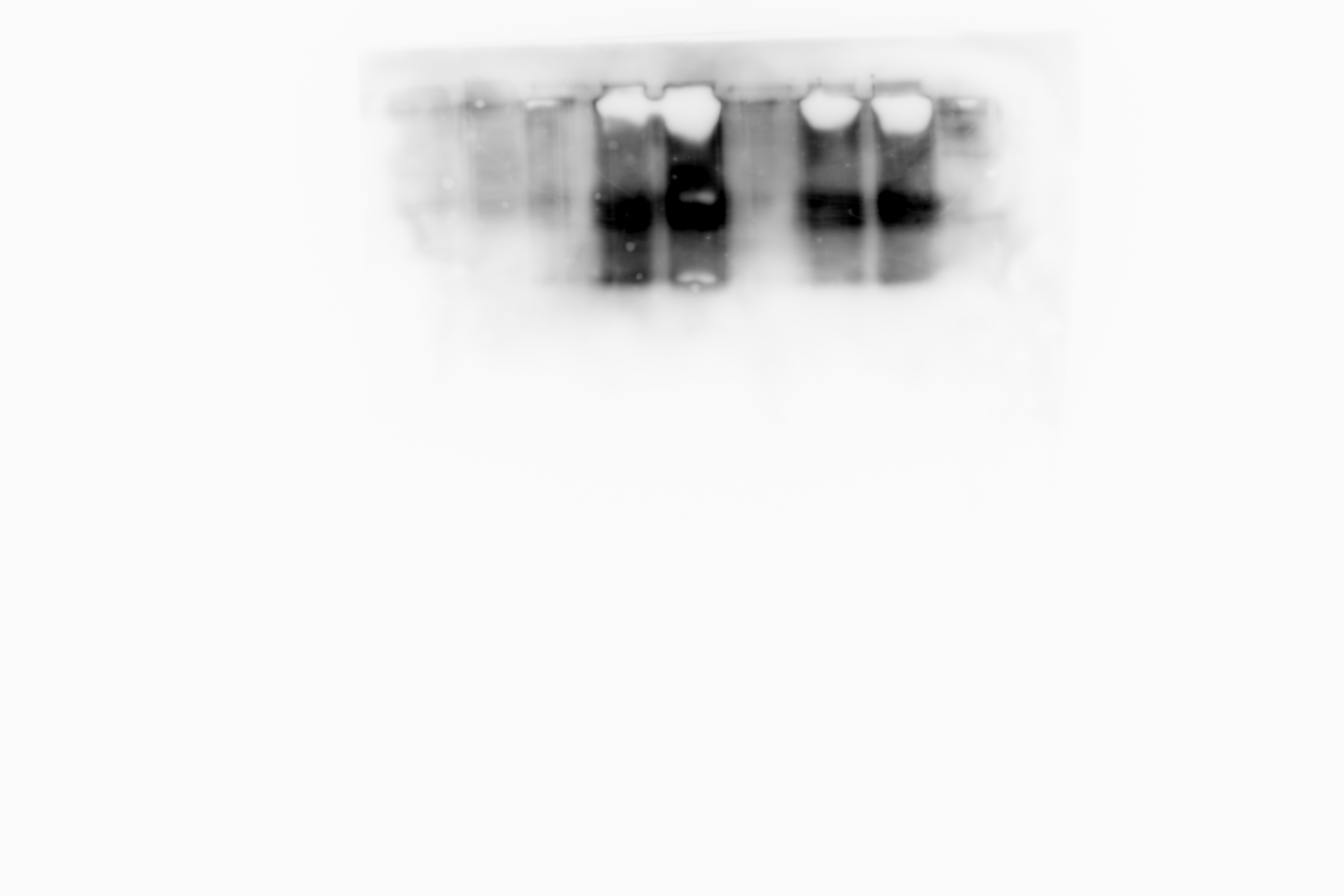

Supplement: Figure 4—source data 1. [file elife-93125-fig4-data1.zip › IP SIRT1_5 (1).tif]

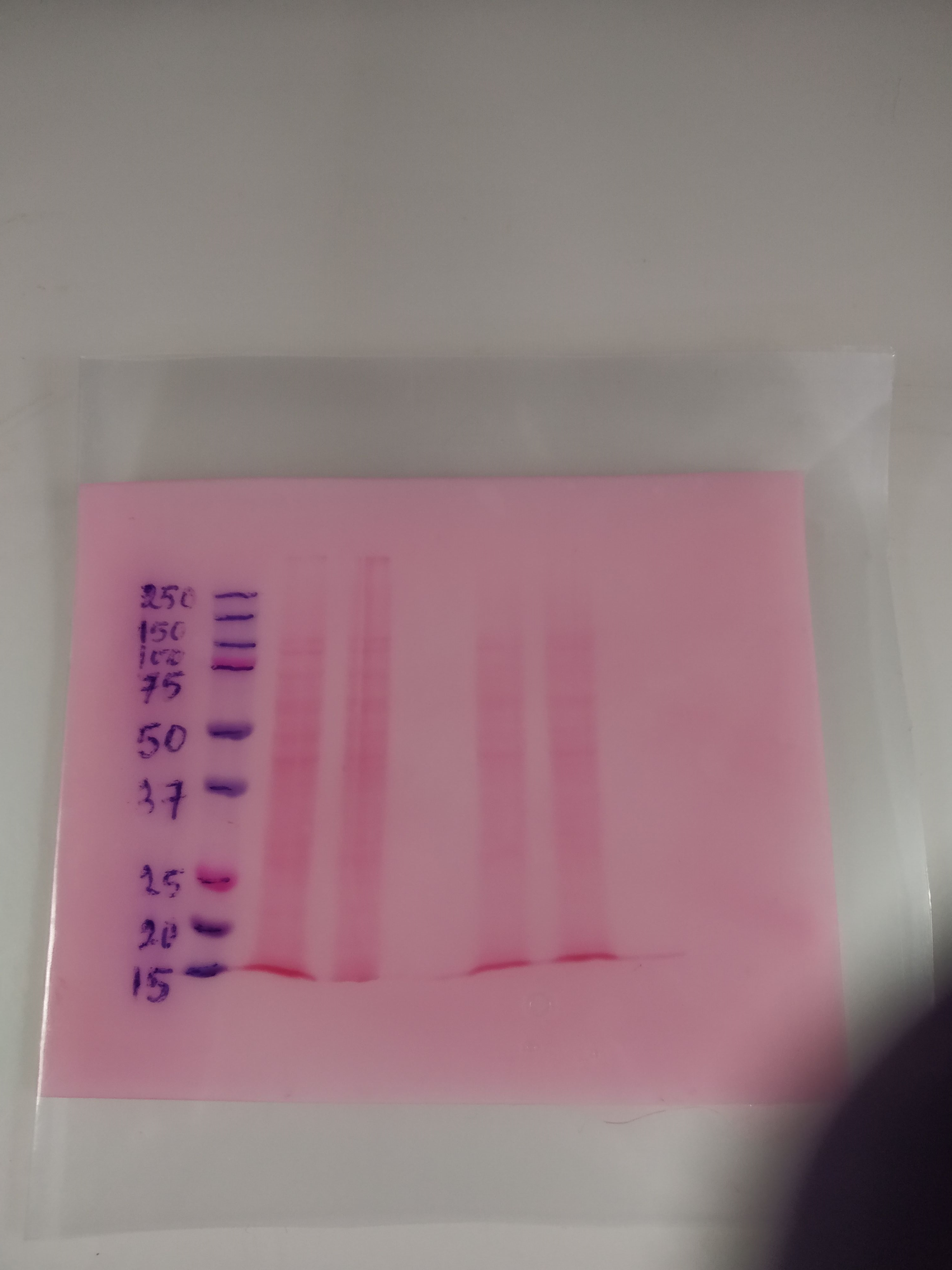

Supplement: Figure 4—source data 1. [file elife-93125-fig4-data1.zip › IMG_20220927_163102.tiff]

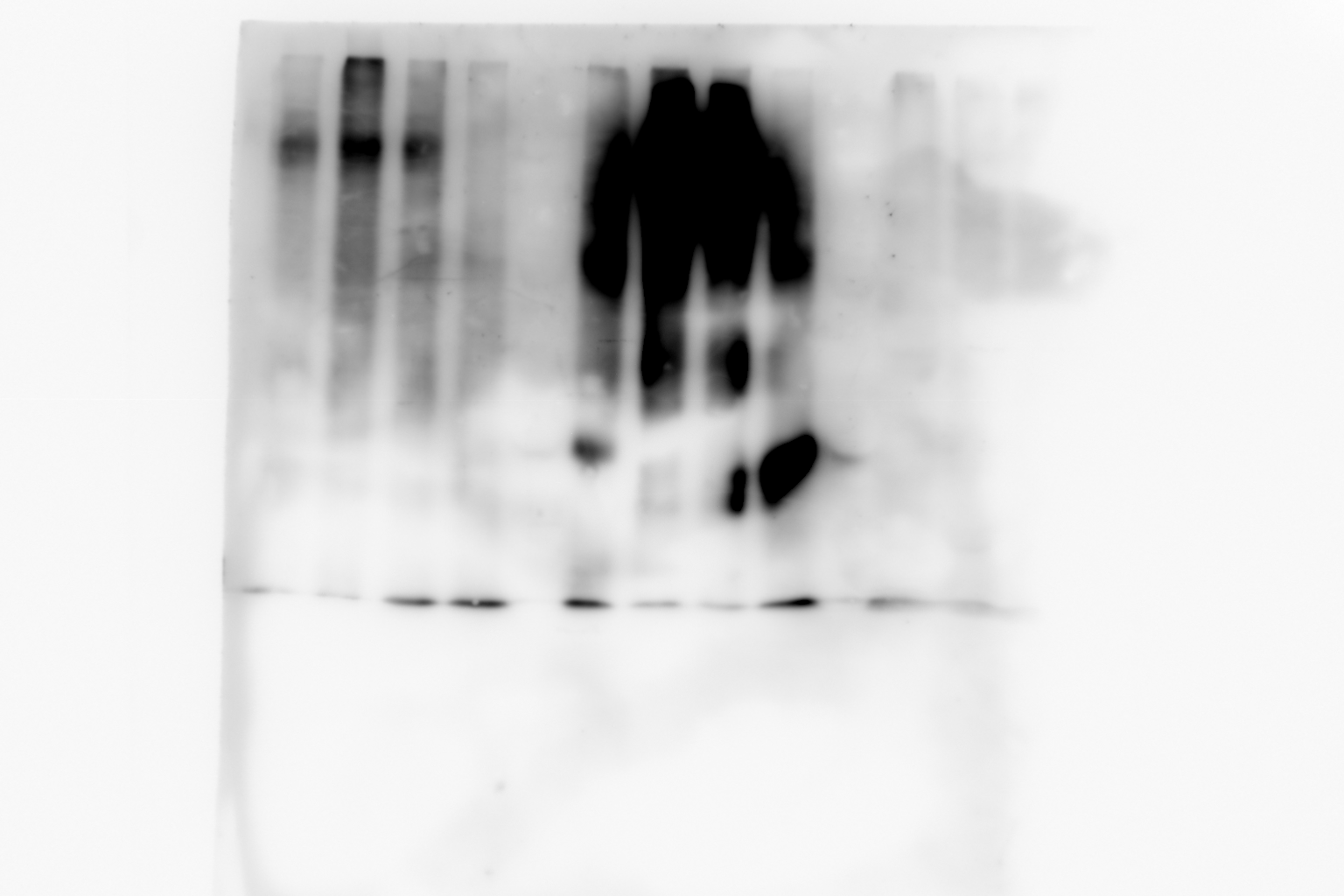

Supplement: Figure 4—source data 2. [file elife-93125-fig4-data2.zip › SIRT1 p65 UT EX poat wash.tif]

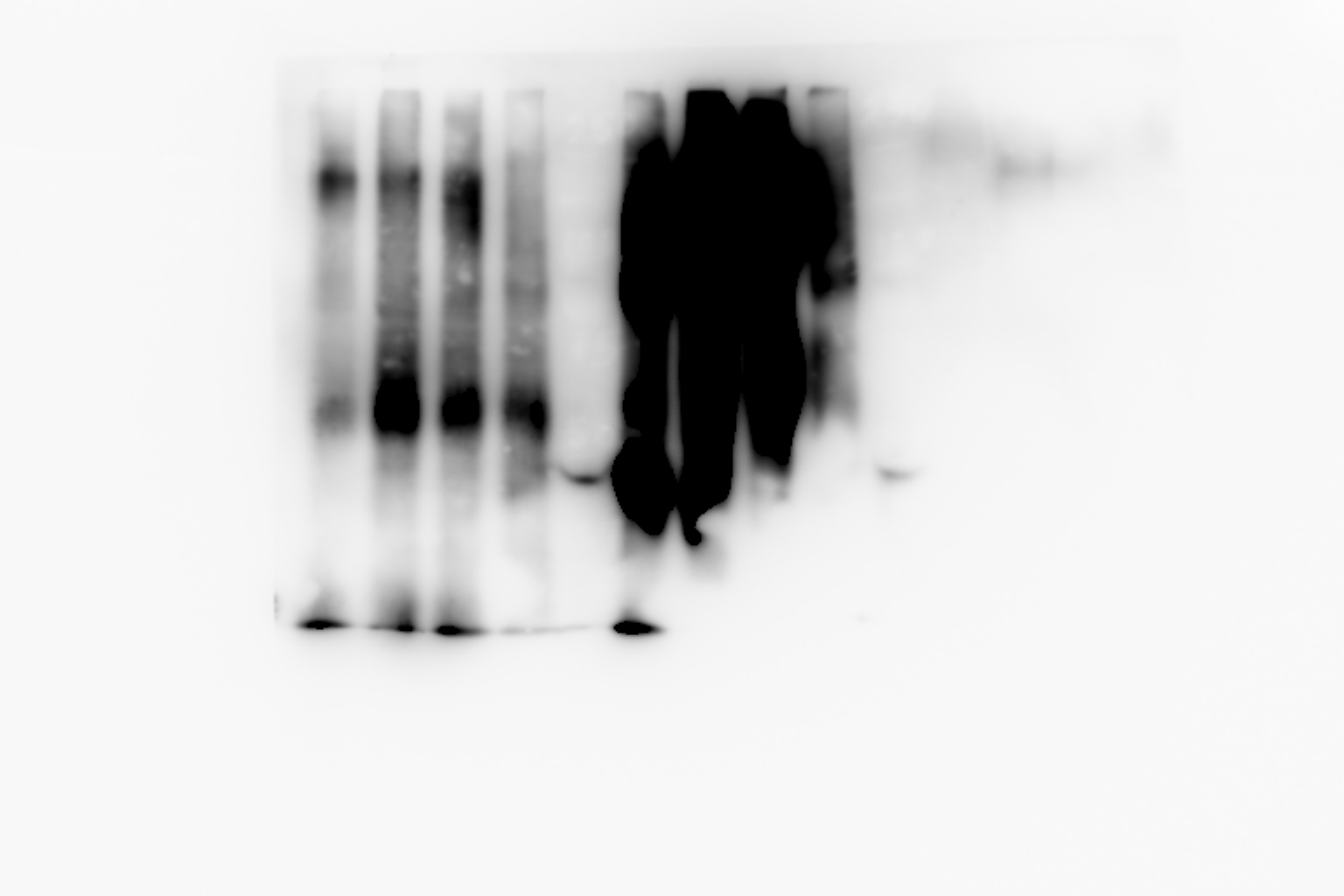

Supplement: Figure 4—source data 2. [file elife-93125-fig4-data2.zip › UI UT EX STM UT STM IP p65.tif]

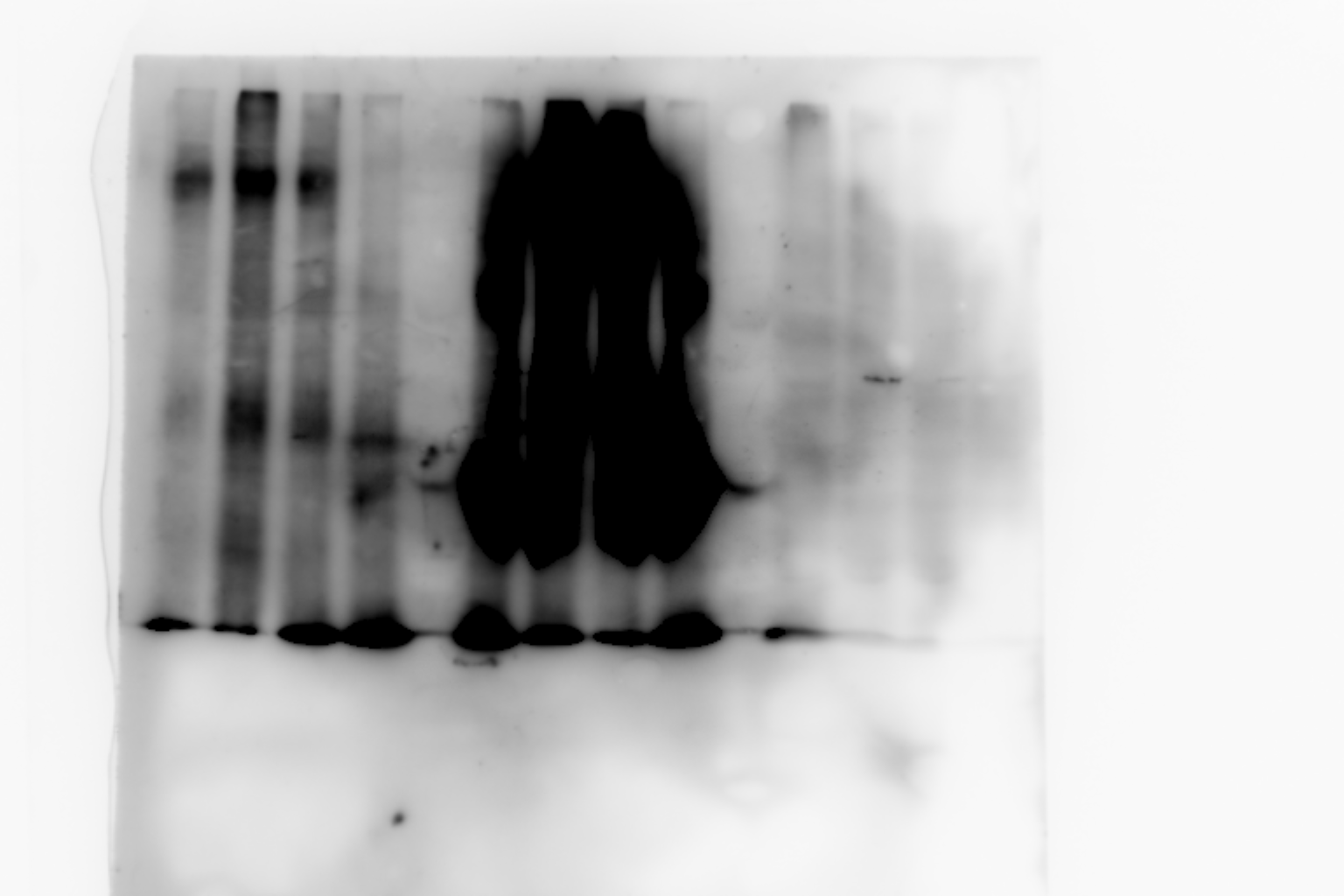

Supplement: Figure 4—source data 2. [file elife-93125-fig4-data2.zip › UI UT EX STM 16h zoomed Ac K p65.tif]

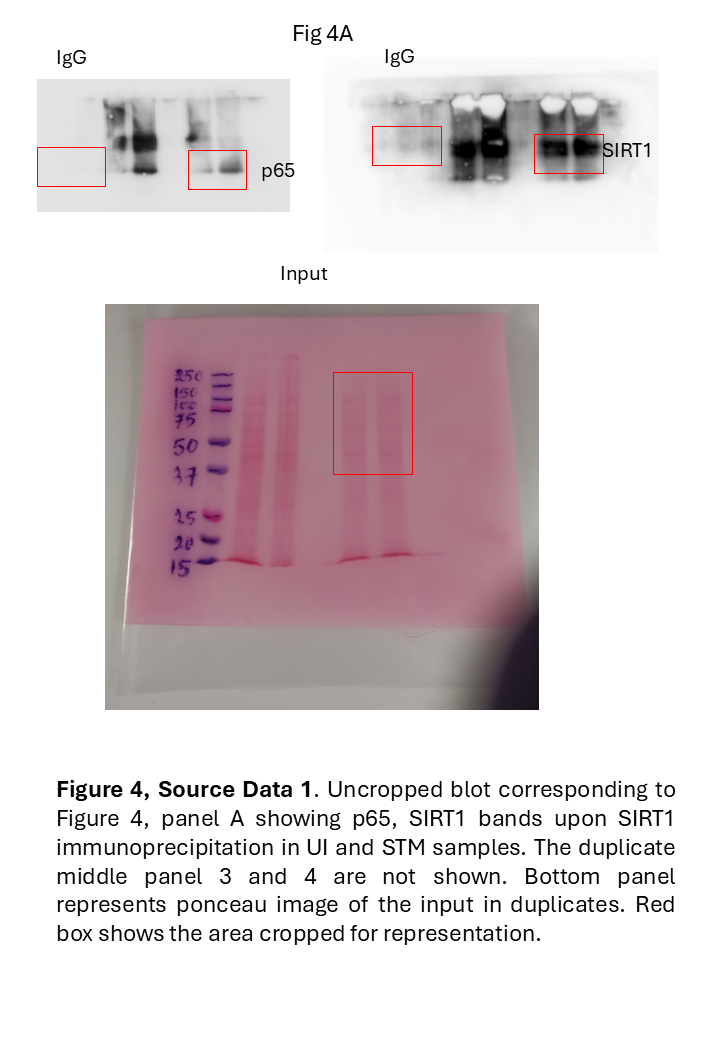

Supplement: Figure 4—source data 4. [file elife-93125-fig4-data4.zip › Figure 4-Source Data 1.TIF]

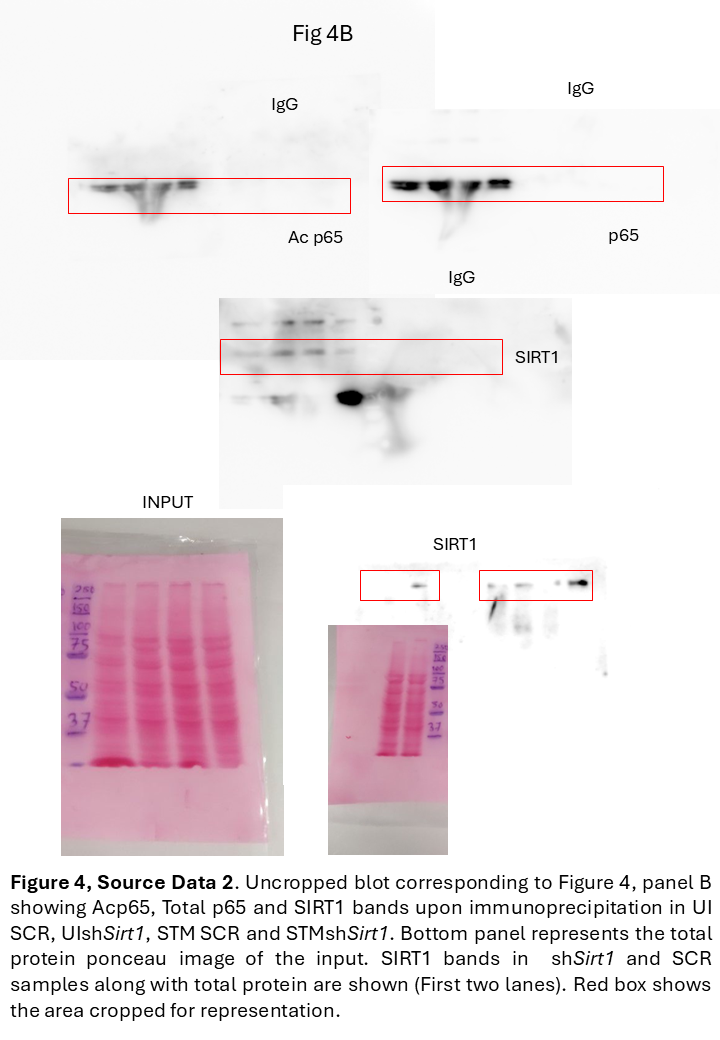

Supplement: Figure 4—source data 5. [file elife-93125-fig4-data5.zip › Figure 4-Source Data 2.TIF]

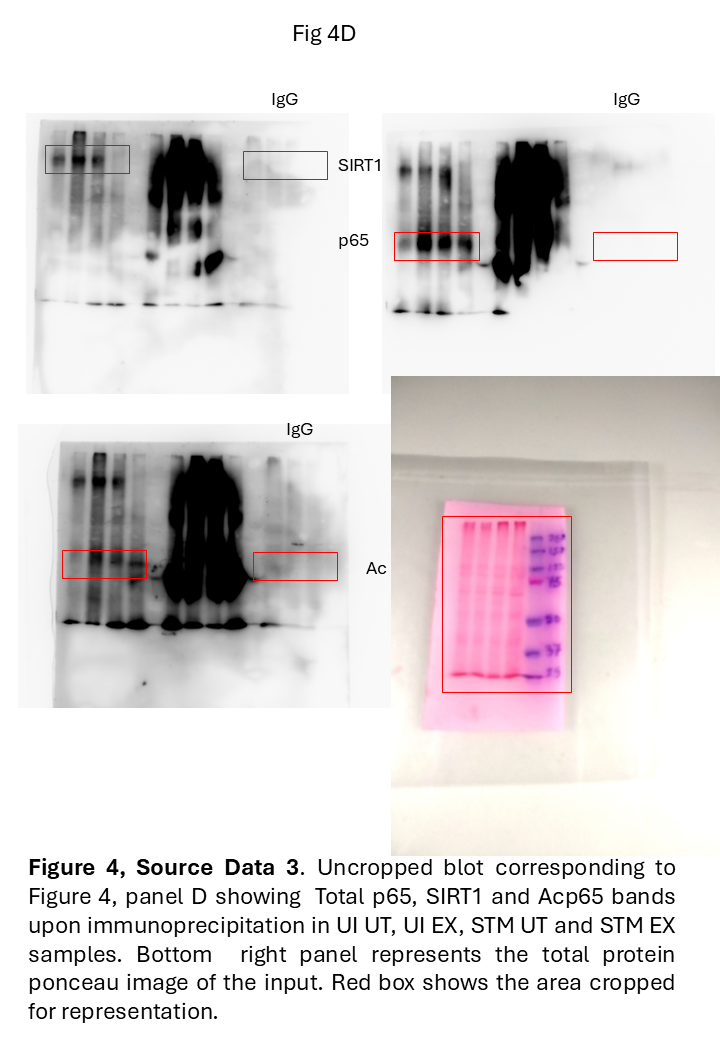

Supplement: Figure 4—source data 6. [file elife-93125-fig4-data6.zip › Figure 4-Source Data 3.TIF]

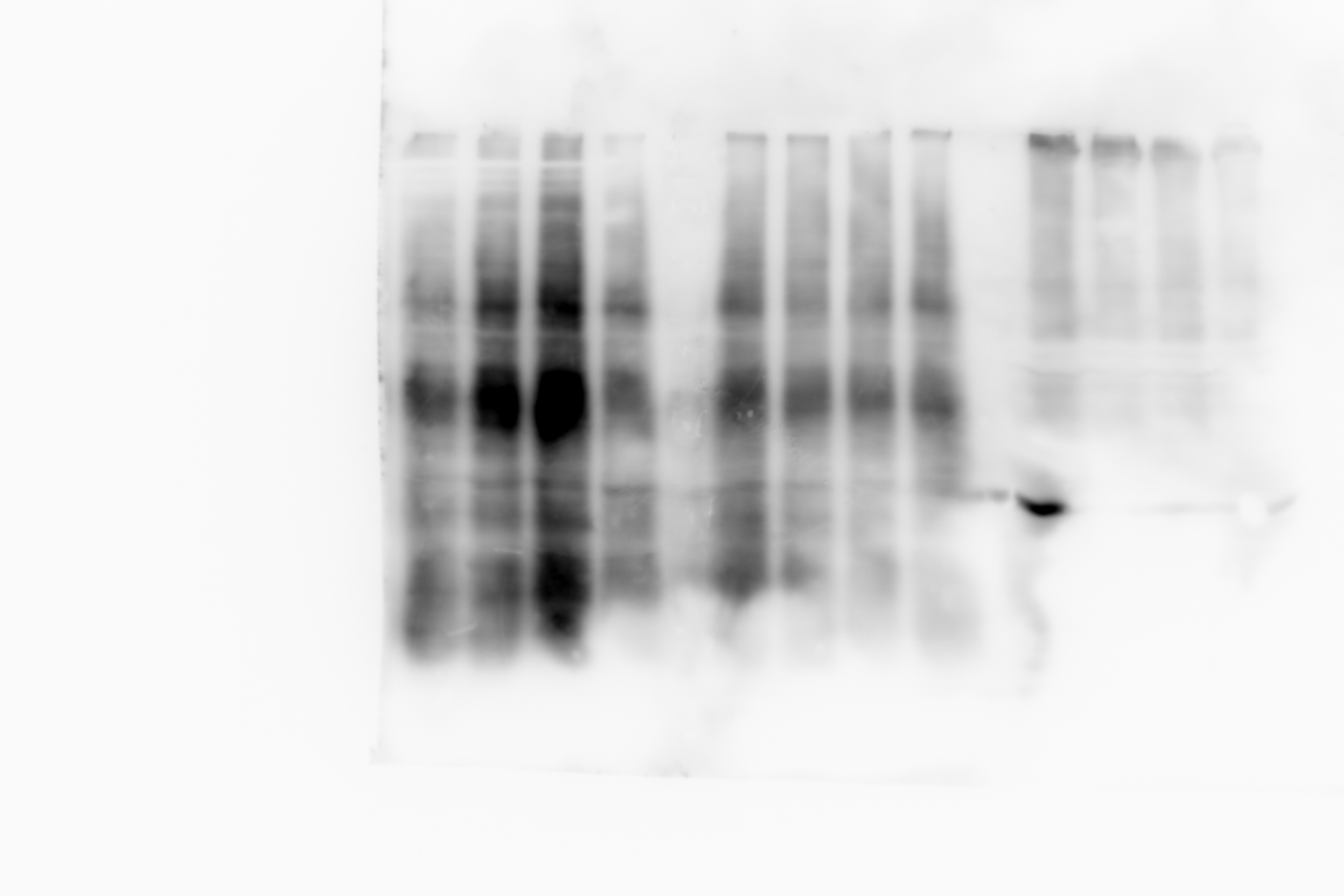

Supplement: Figure 7—source data 3. [file elife-93125-fig7-data3.zip › UT EX UI STM UT EX SIRT1 HIF1 IP.tif]

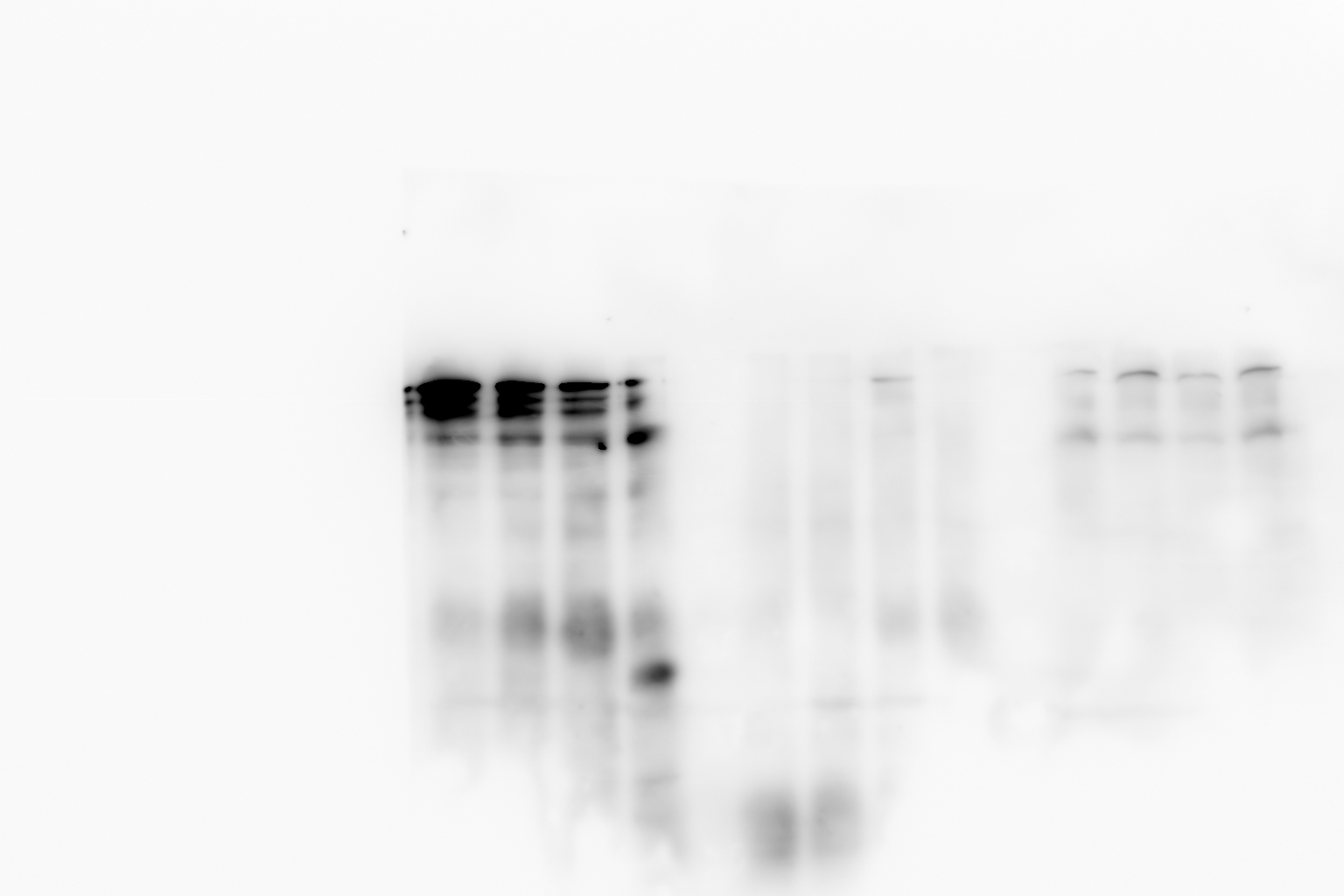

Supplement: Figure 7—source data 3. [file elife-93125-fig7-data3.zip › UI UT EX STM UT EX 16h HIF control ft zoomed_22 - Copy.tif]

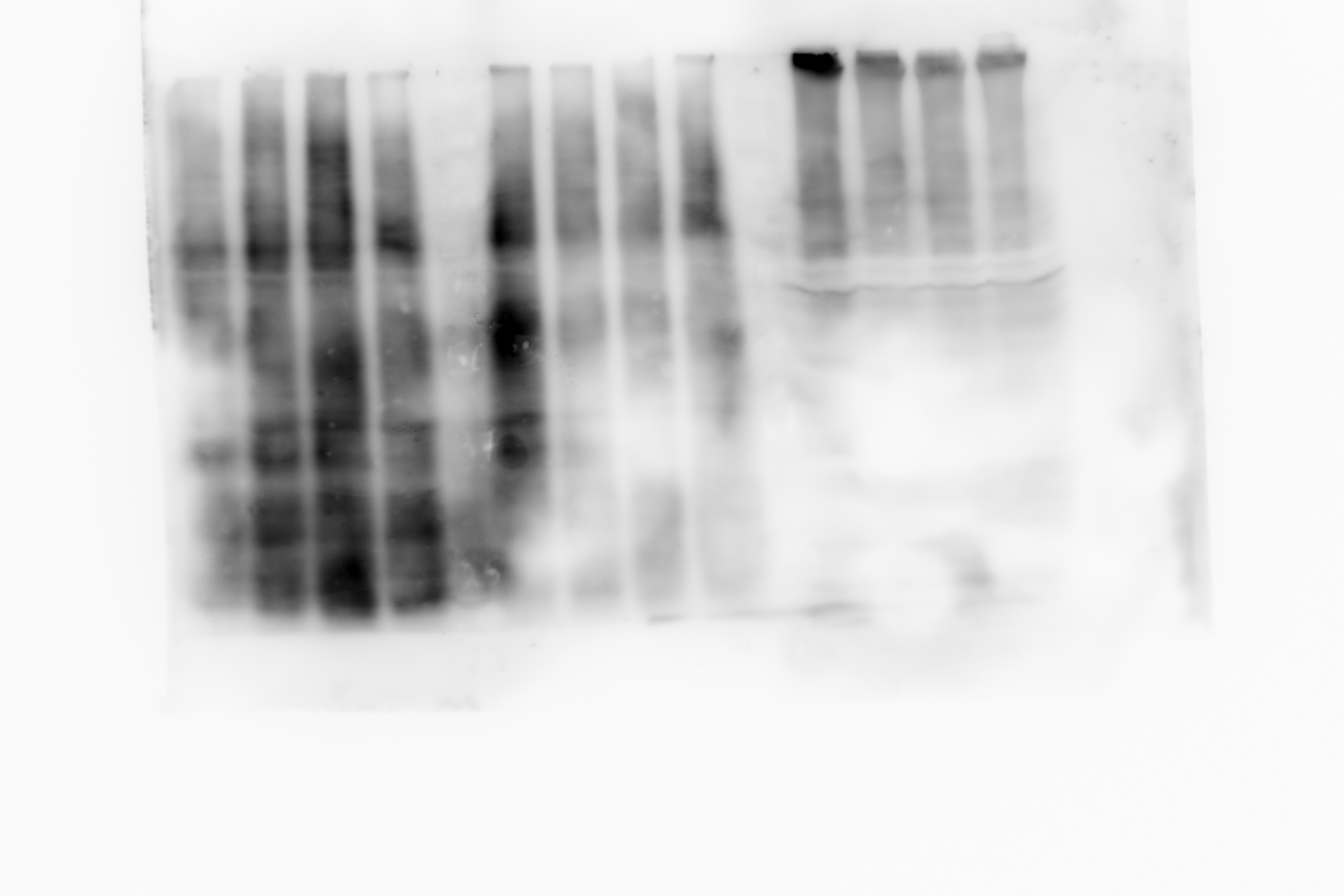

Supplement: Figure 7—source data 3. [file elife-93125-fig7-data3.zip › UI UT EX STM UT EX zoomed_13 Ac HIF1.tif]

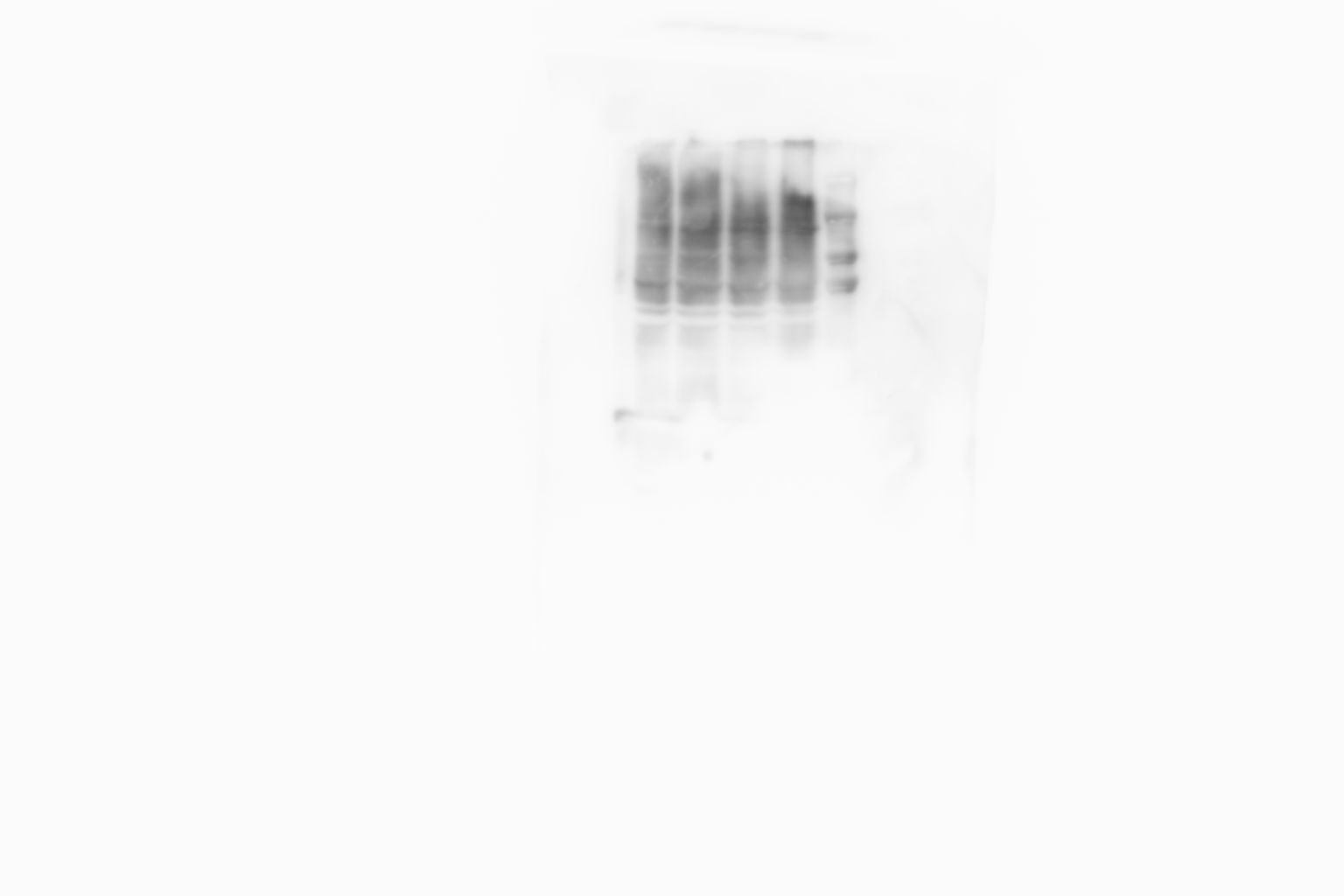

Supplement: Figure 7—source data 3. [file elife-93125-fig7-data3.zip › 20220712_1456_2SIRT1 inh HIF IP input HIF1 a.tif]

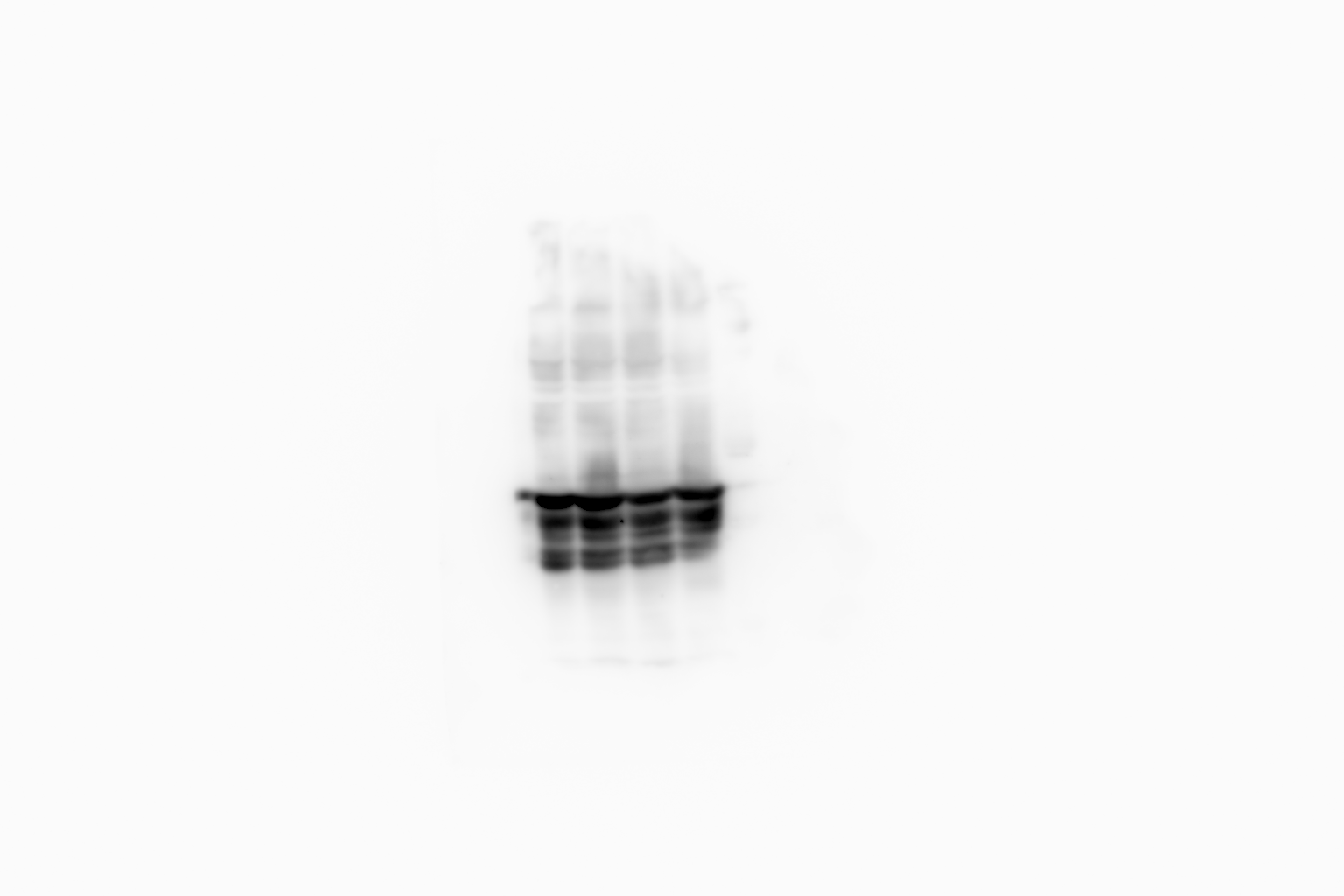

Supplement: Figure 7—source data 3. [file elife-93125-fig7-data3.zip › UI STM UT EX 16h_2.tif]

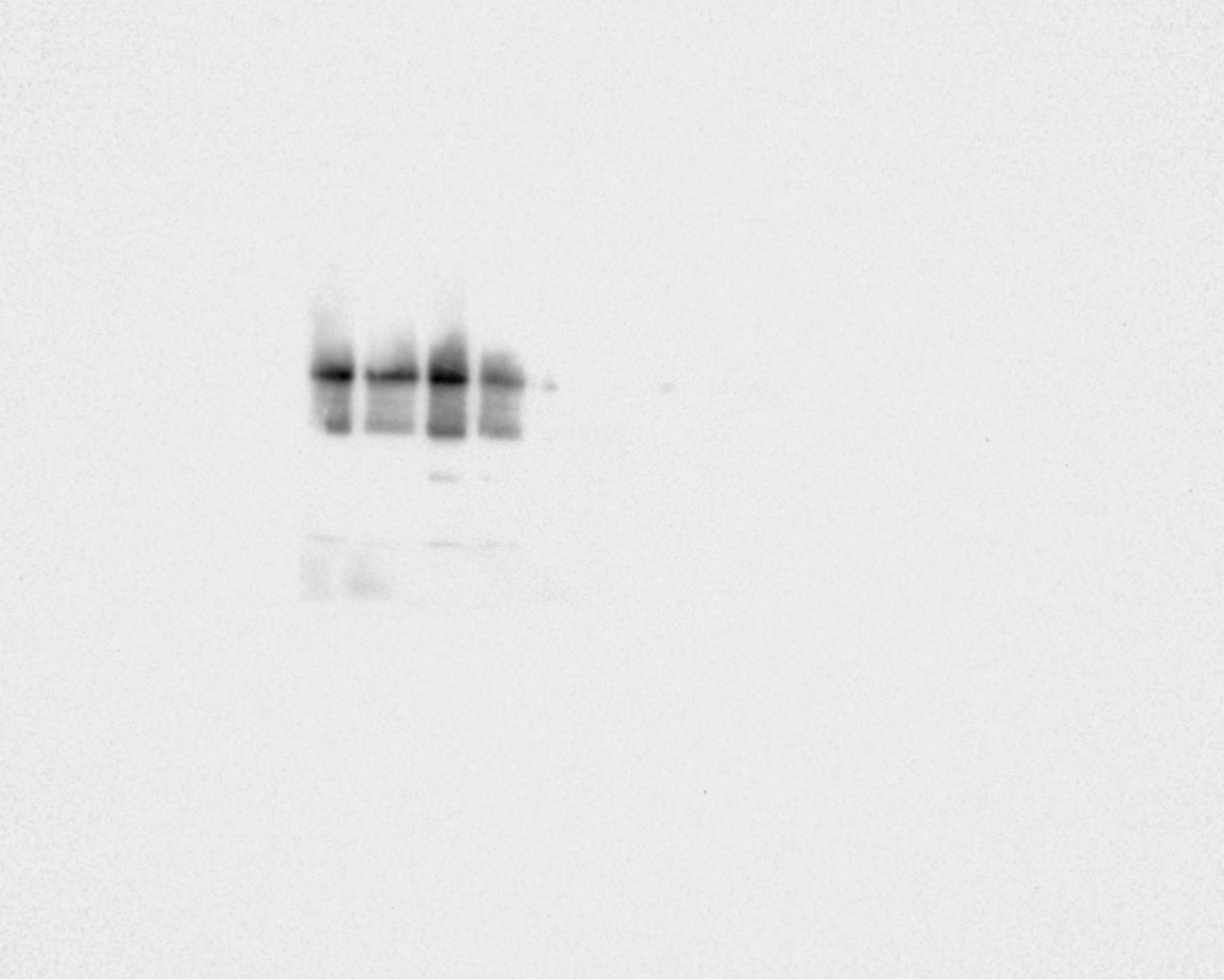

Supplement: Figure 7—source data 4. [file elife-93125-fig7-data4.zip › SIRT3 PDHA1IP UI STM SCR shSirt32024-07-12 16h31m50s(Chemiluminescence).tif]

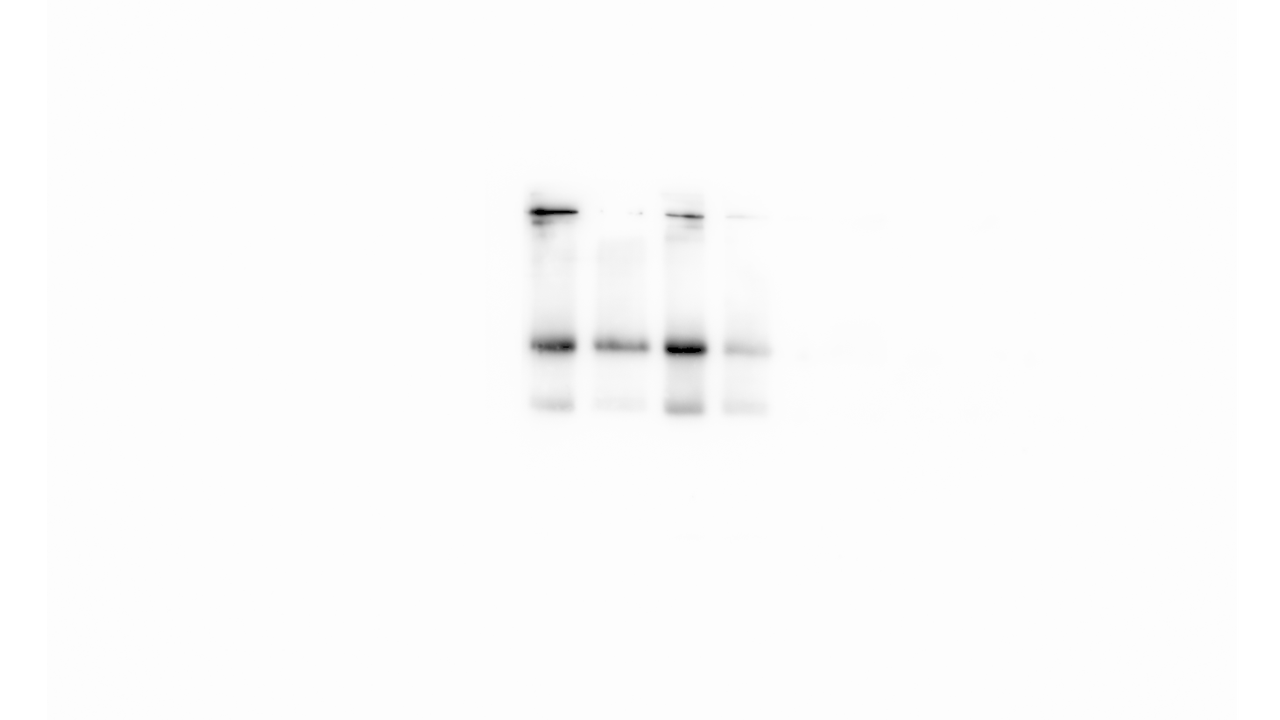

Supplement: Figure 7—source data 4. [file elife-93125-fig7-data4.zip › PDHA1 IP.tif]

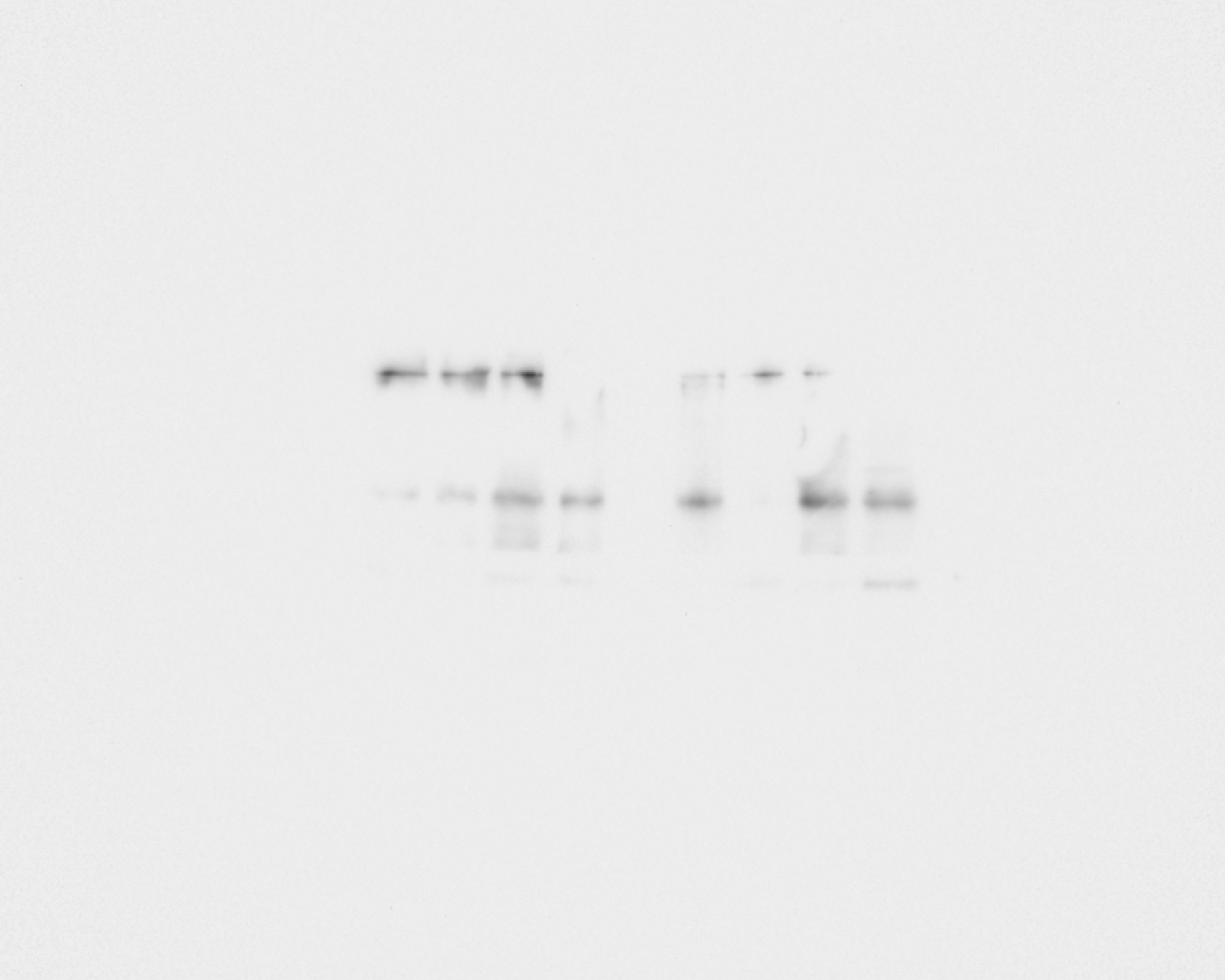

Supplement: Figure 7—source data 4. [file elife-93125-fig7-data4.zip › SIRT3 Input DH DC Lab 2024-07-16 14h24m56s(Chemiluminescence).tif]

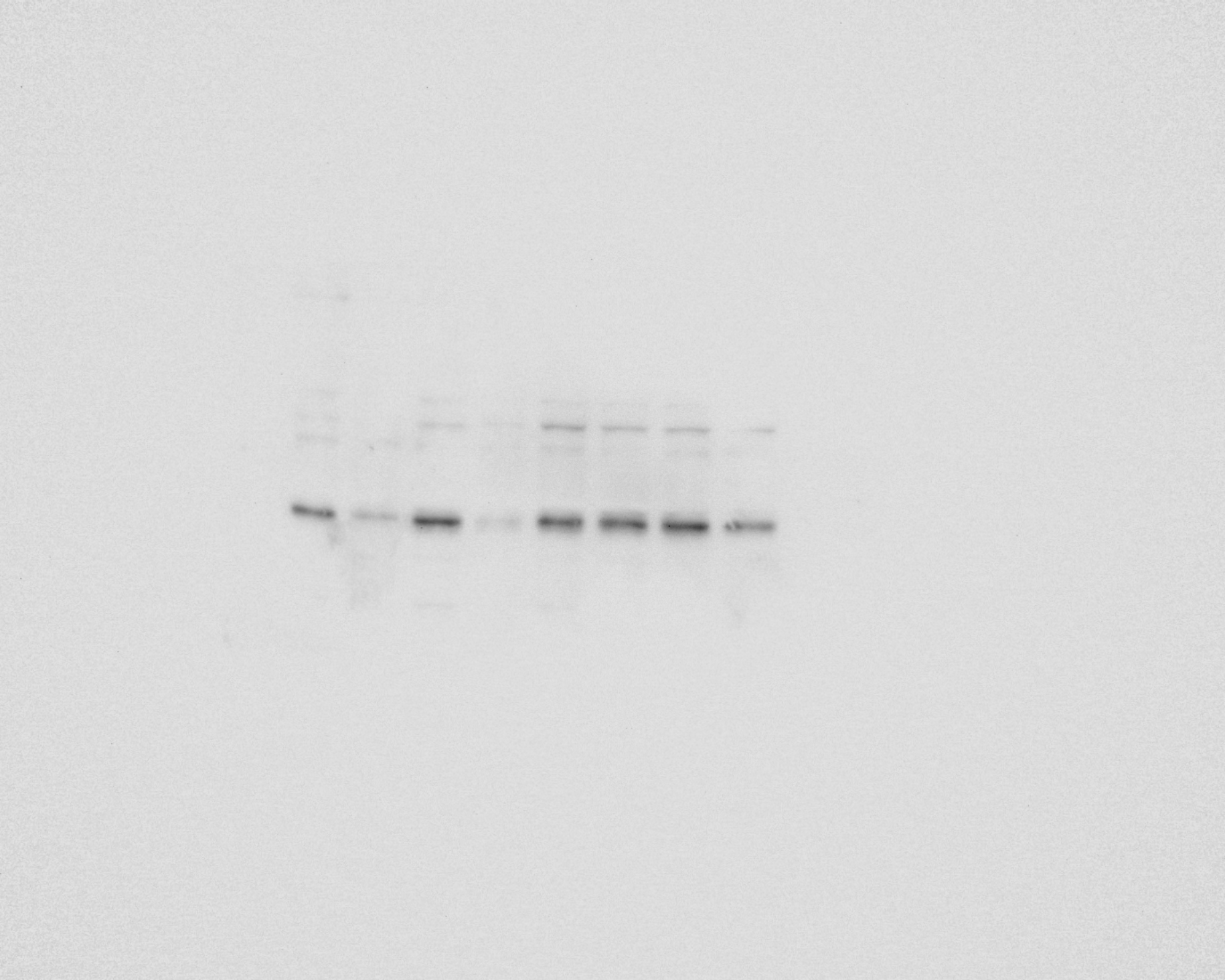

Supplement: Figure 7—source data 4. [file elife-93125-fig7-data4.zip › pdha1 input_6(Chemiluminescence).tif]

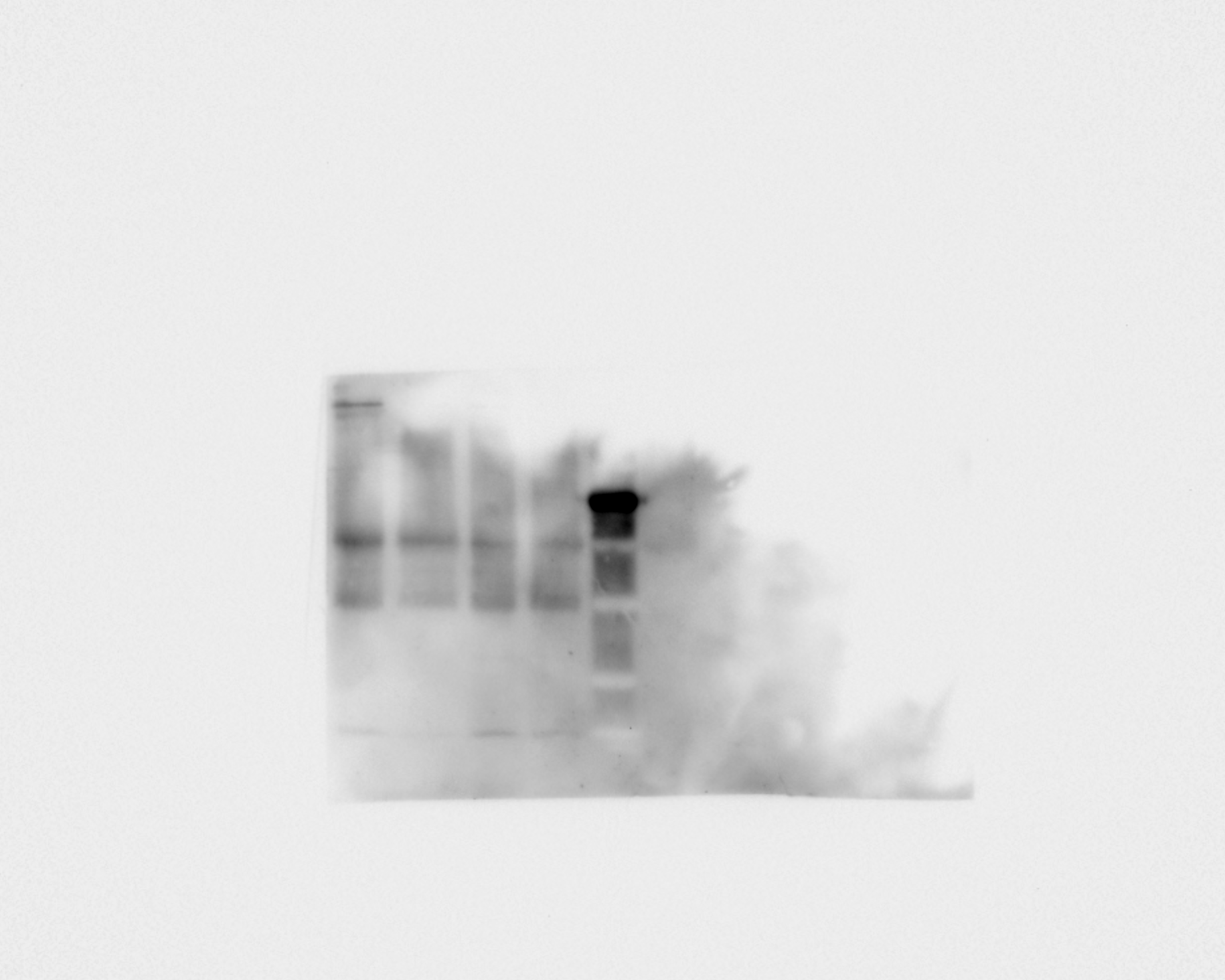

Supplement: Figure 7—source data 4. [file elife-93125-fig7-data4.zip › ack sirt3 kd pdha1_5(Chemiluminescence).tif]

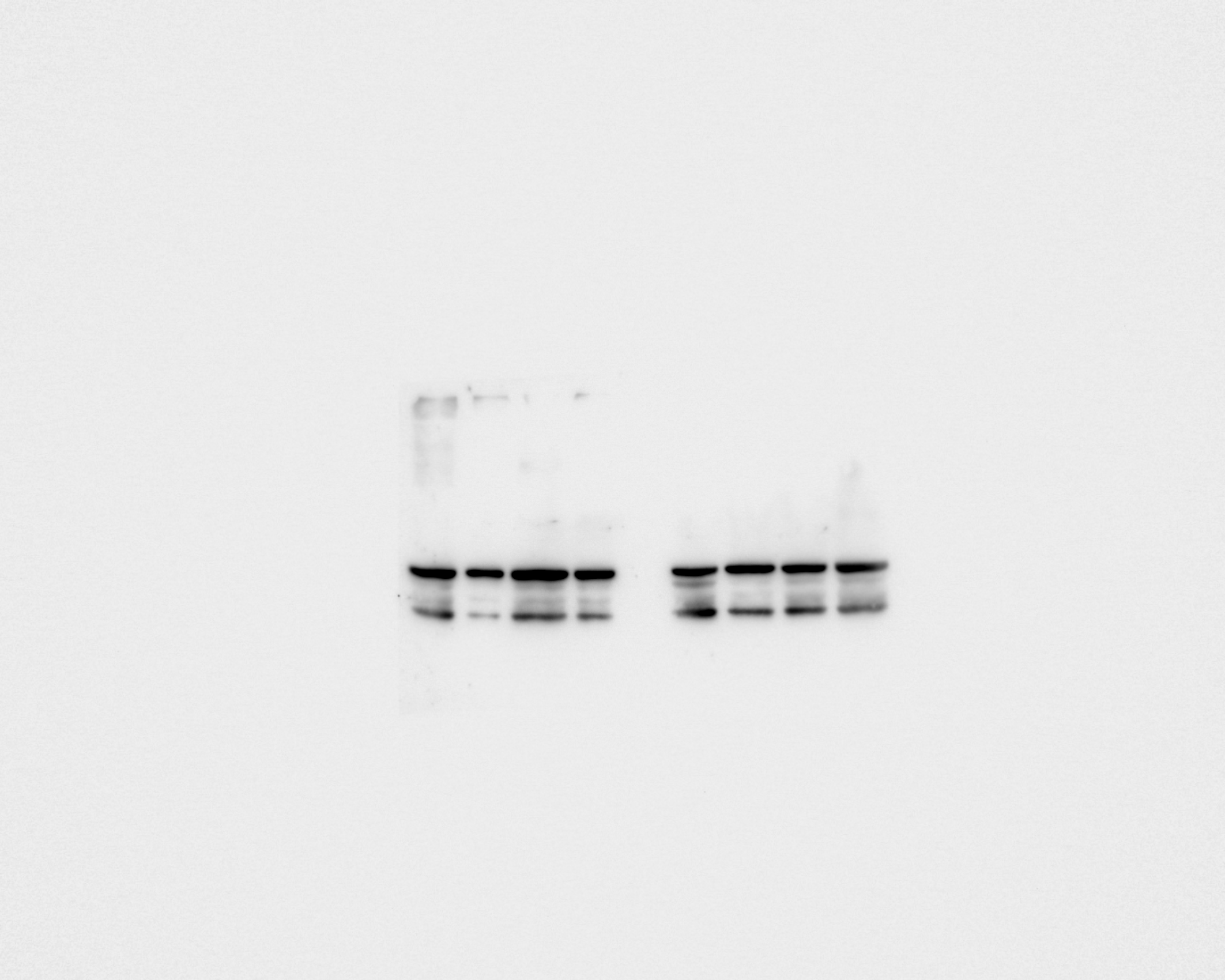

Supplement: Figure 7—source data 4. [file elife-93125-fig7-data4.zip › dh 2024-07-12 16h42m36s(Chemiluminescence).tif]

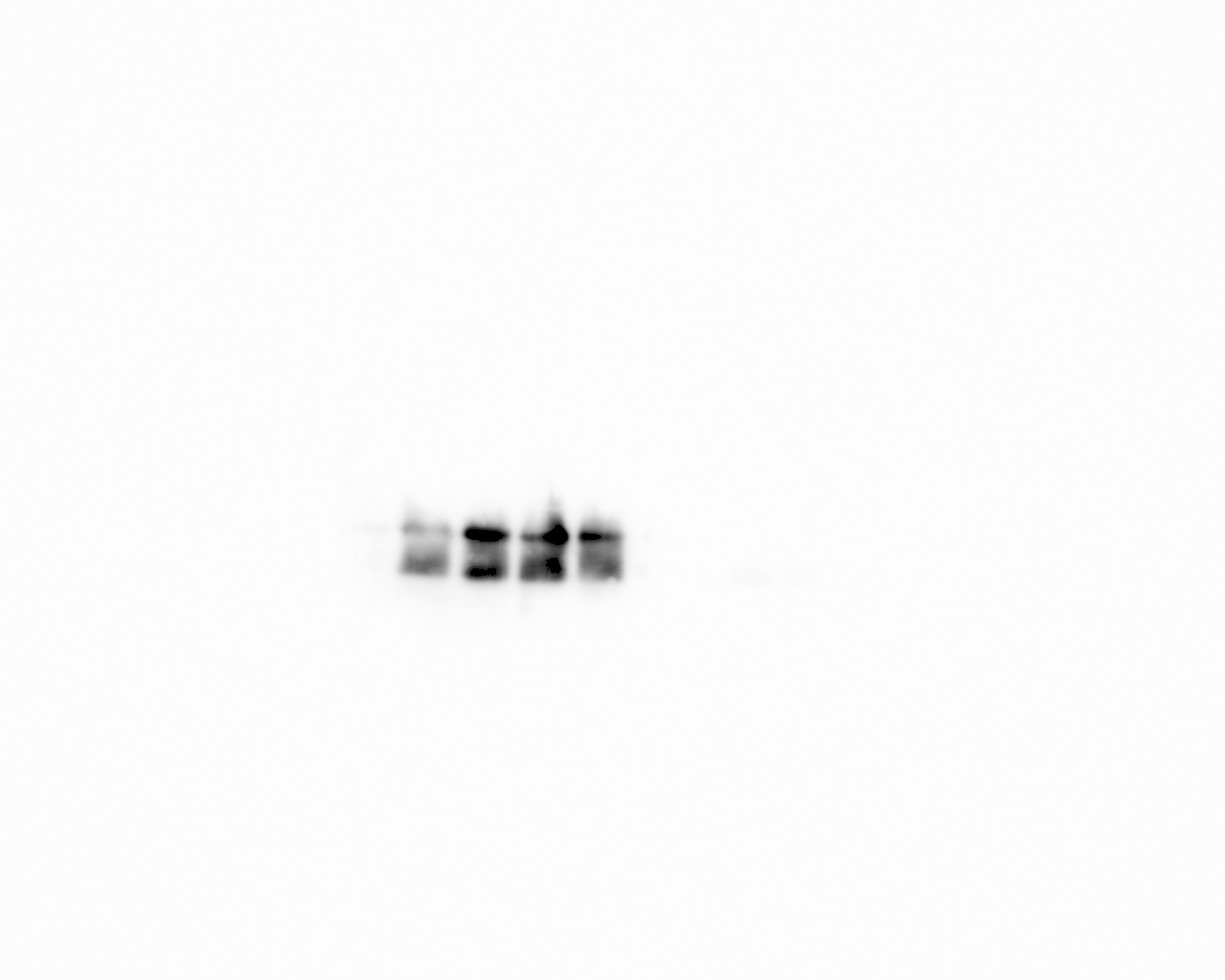

Supplement: Figure 7—source data 5. [file elife-93125-fig7-data5.zip › PDHA1 ip SIRT3 dh 2024-02-23 14h47m42s(Chemiluminescence).raw16.tif]

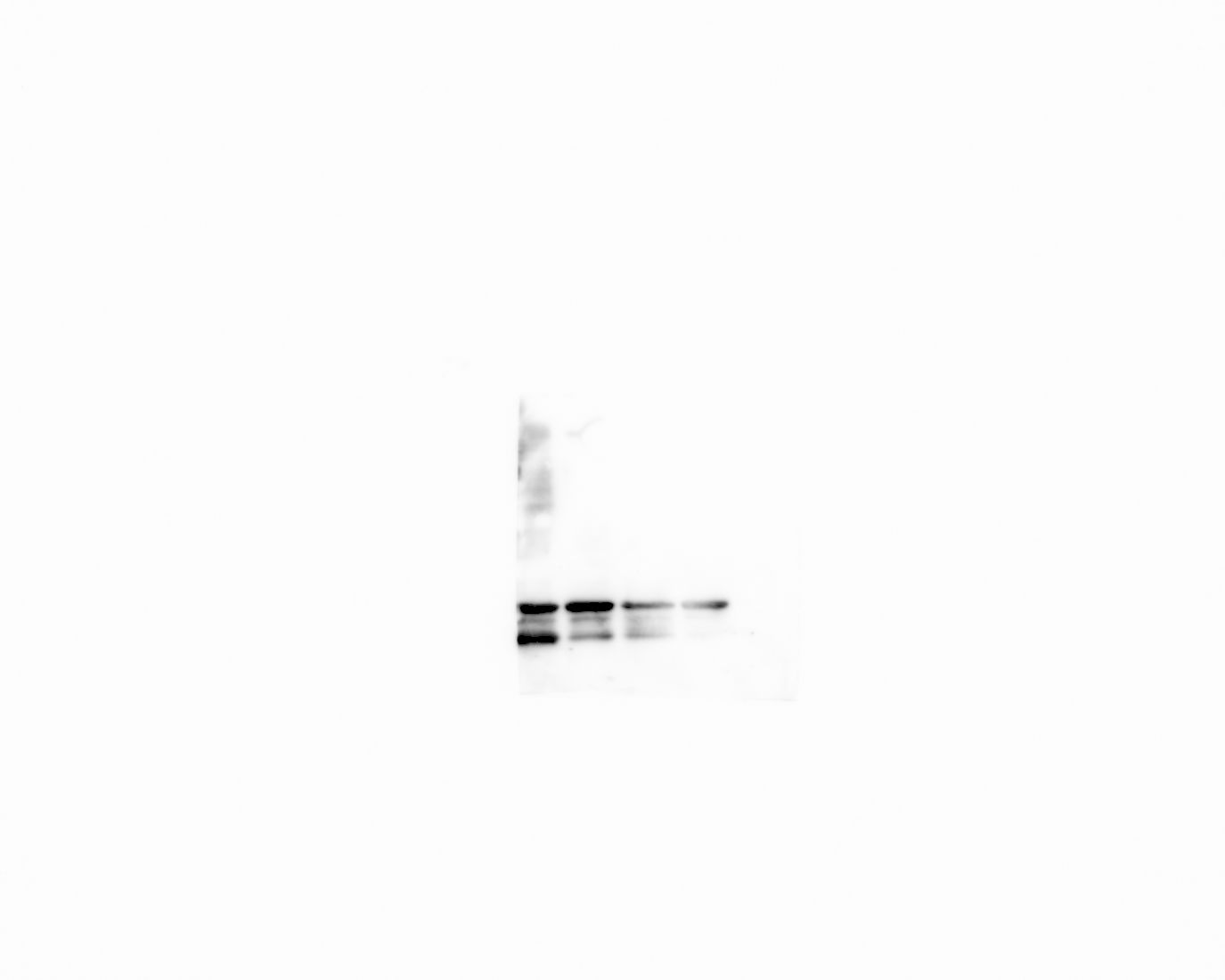

Supplement: Figure 7—source data 5. [file elife-93125-fig7-data5.zip › b actin ft_05(Chemiluminescence).raw16.tif]

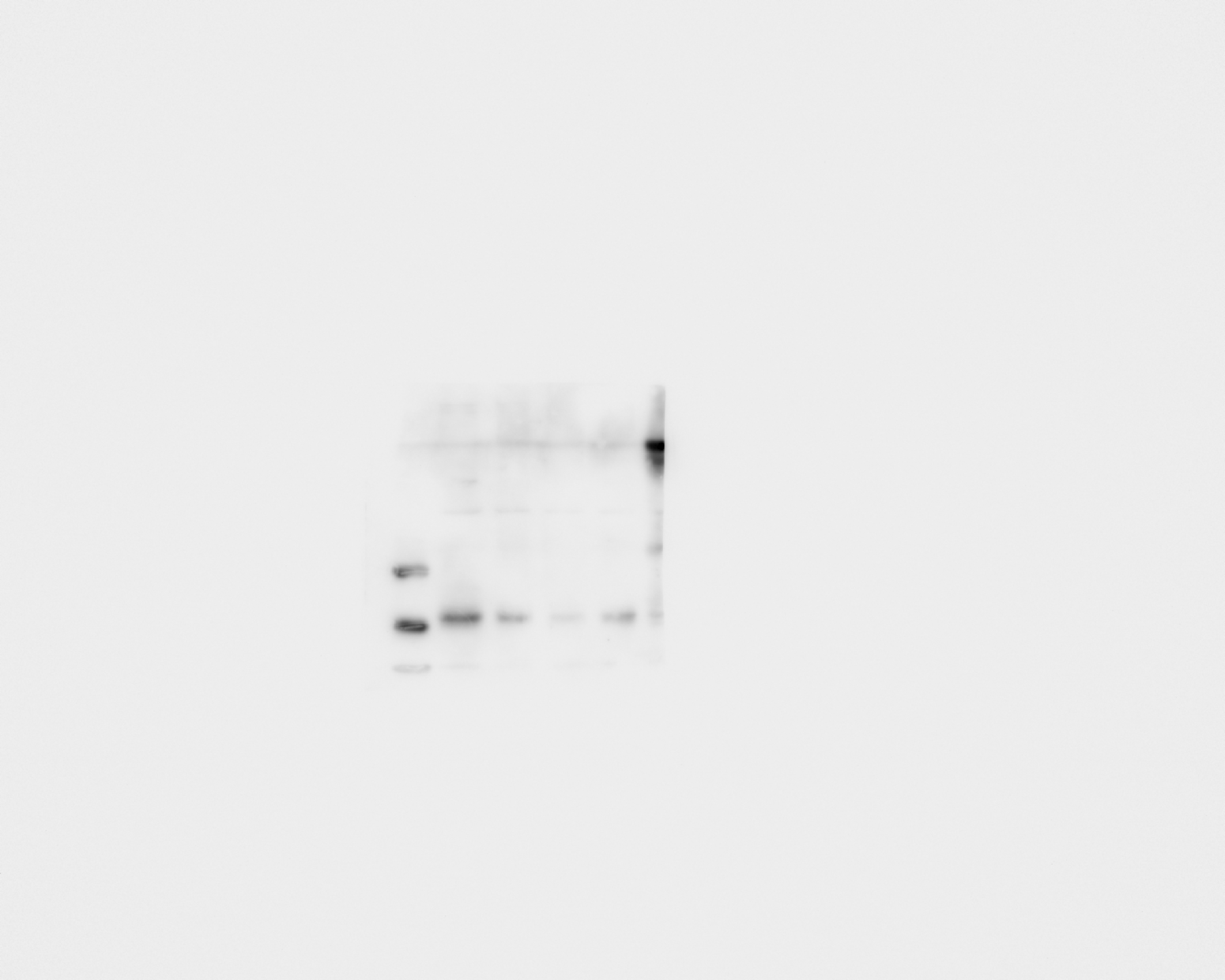

Supplement: Figure 7—source data 5. [file elife-93125-fig7-data5.zip › pdha1 input_05(Chemiluminescence).tif]

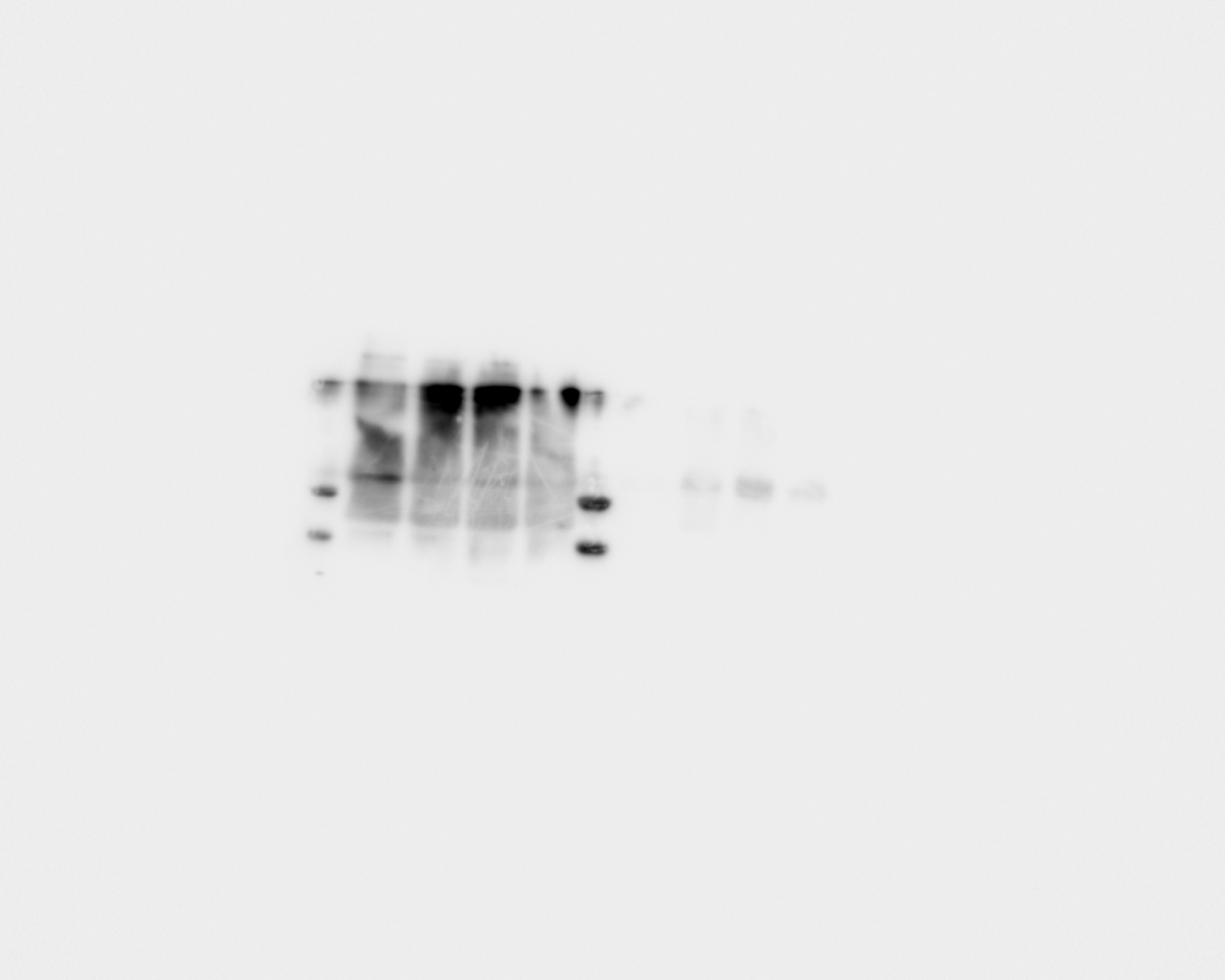

Supplement: Figure 7—source data 5. [file elife-93125-fig7-data5.zip › pdha ip pdha_03(Chemiluminescence).tif]

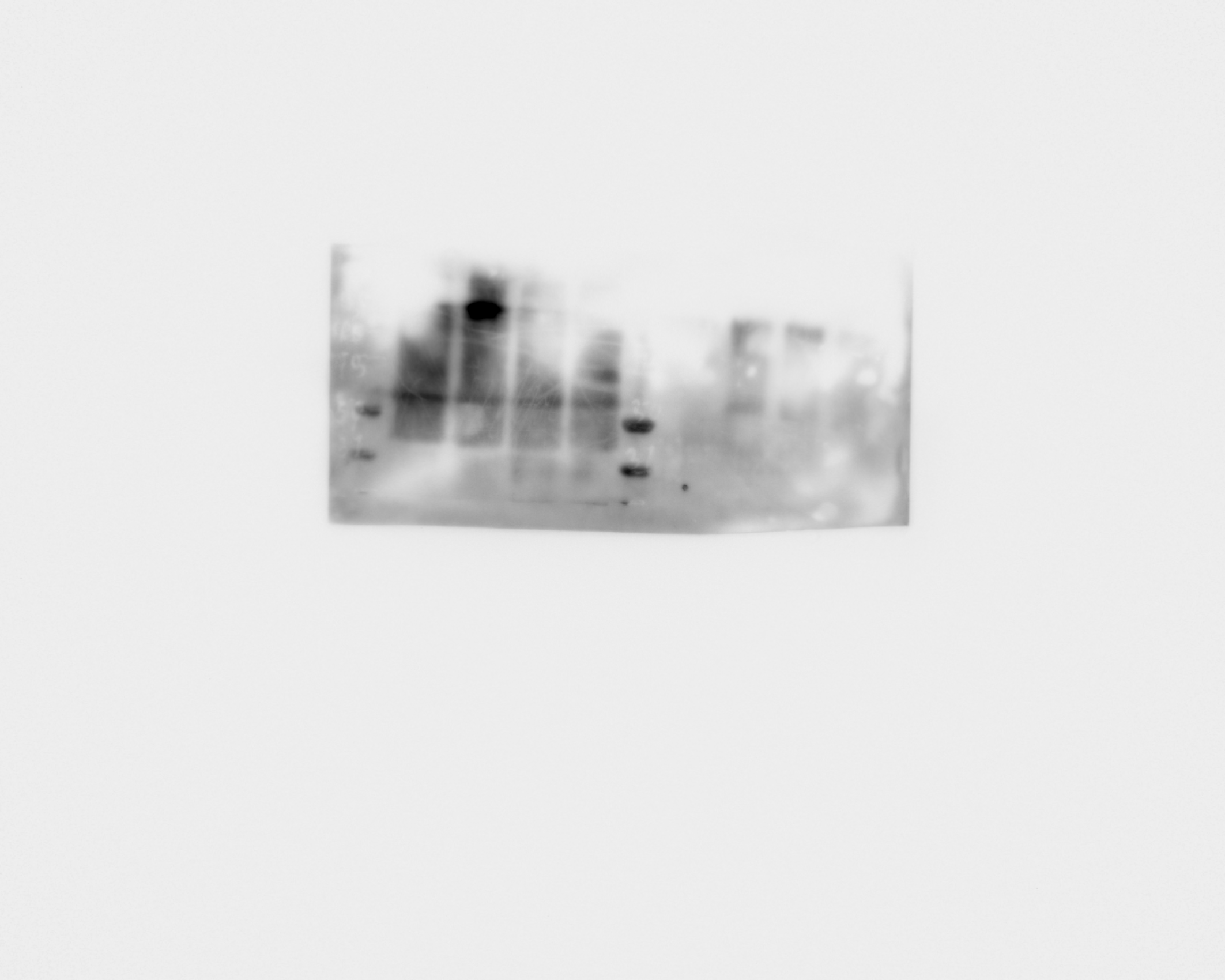

Supplement: Figure 7—source data 5. [file elife-93125-fig7-data5.zip › acK pdha1_13(Chemiluminescence).tif]

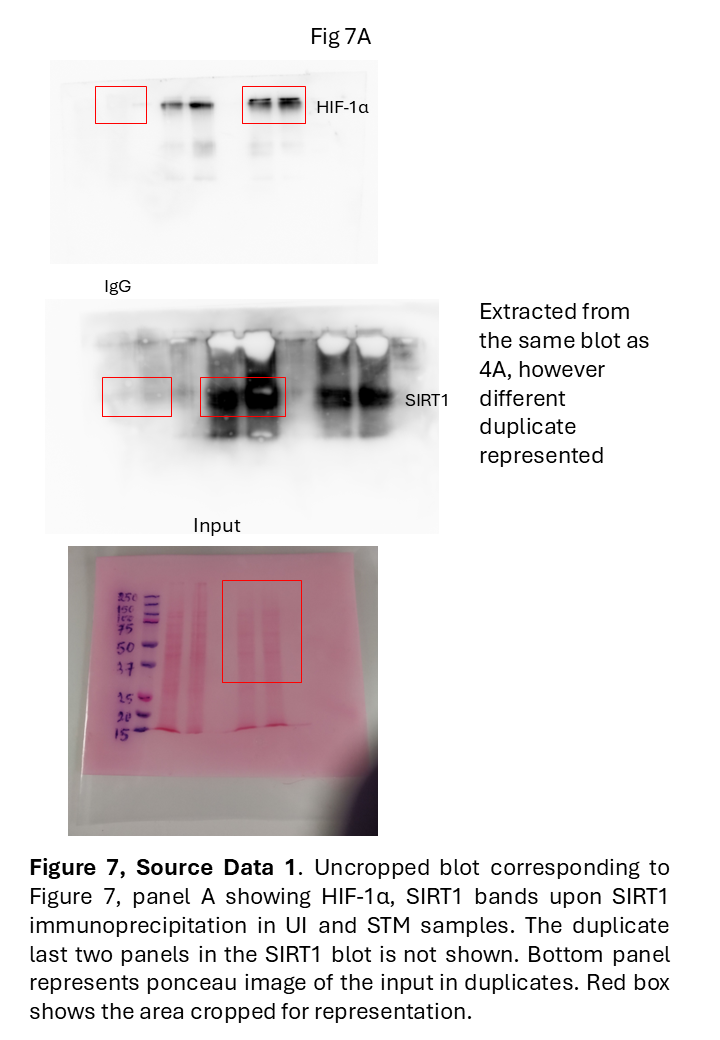

Supplement: Figure 7—source data 6. [file elife-93125-fig7-data6.zip › Figure 7-Source Data 1.TIF]

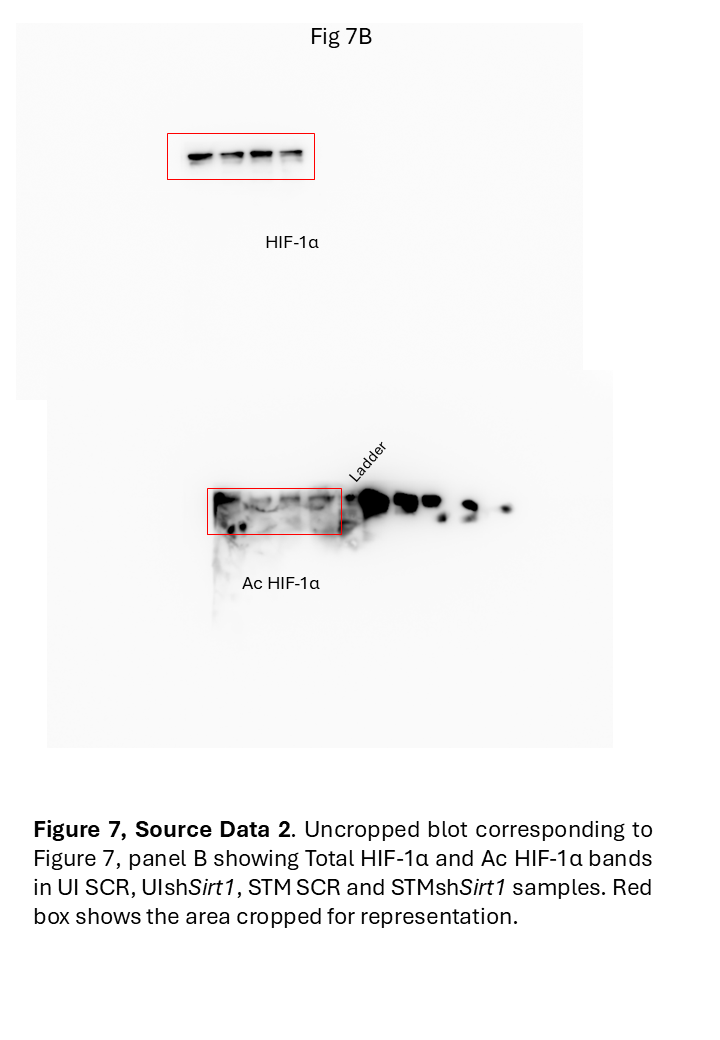

Supplement: Figure 7—source data 7. [file elife-93125-fig7-data7.zip › Figure 7-Source Data 2.TIF]

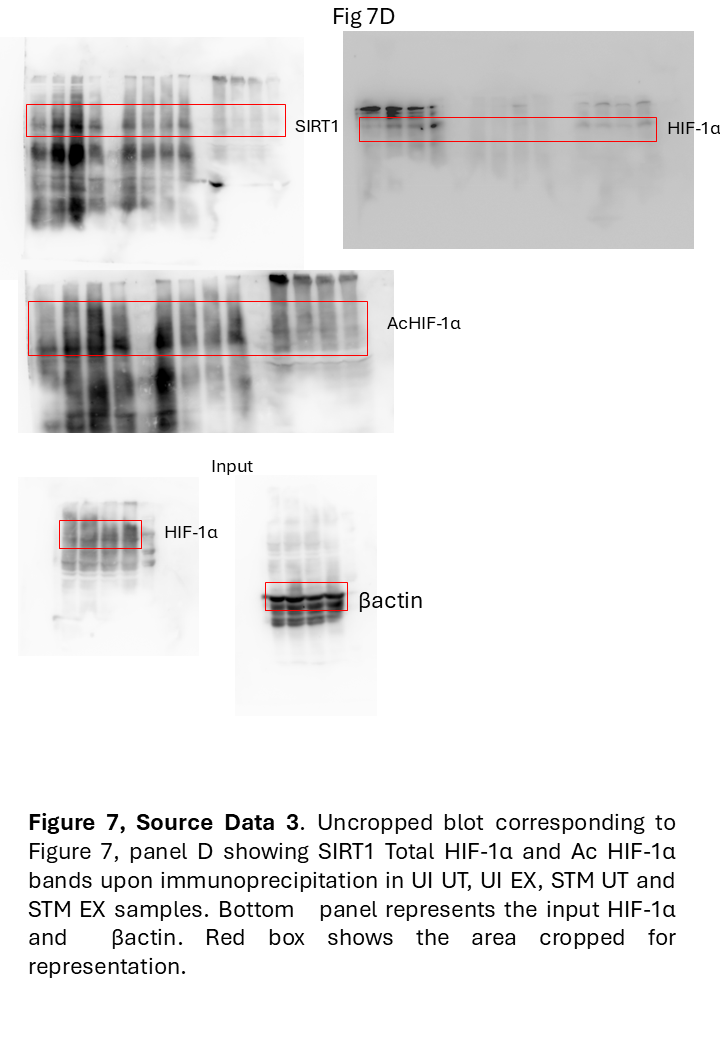

Supplement: Figure 7—source data 8. [file elife-93125-fig7-data8.zip › Figure 7-Source Data 3.TIF]

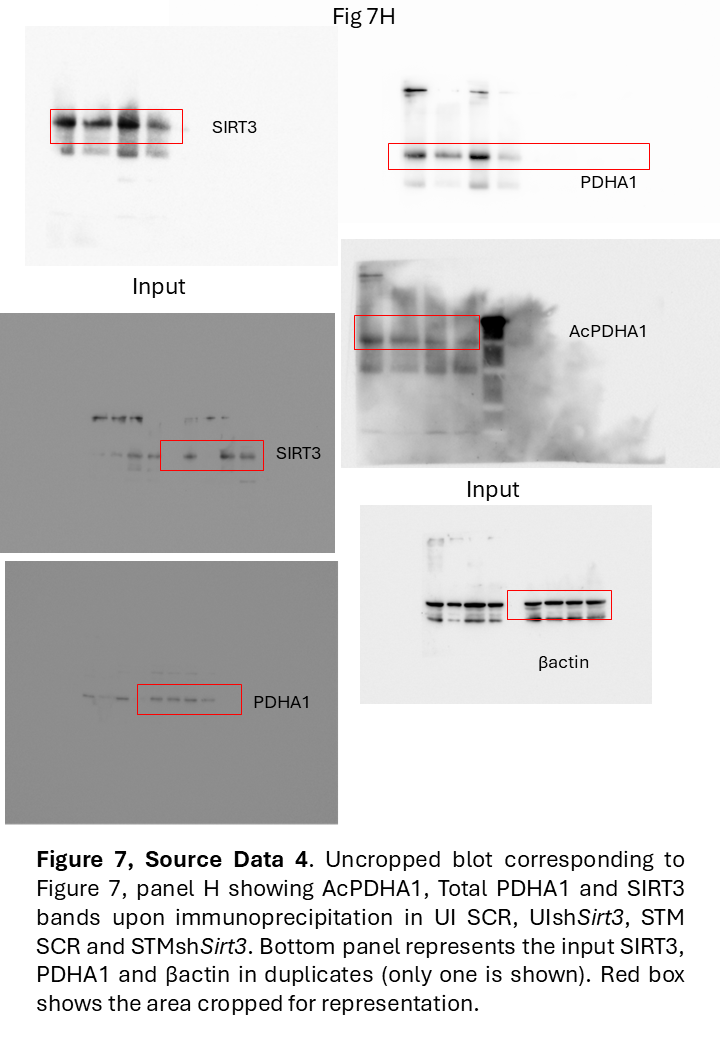

Supplement: Figure 7—source data 9. [file elife-93125-fig7-data9.zip › Figure 7-Source Data 4.TIF]

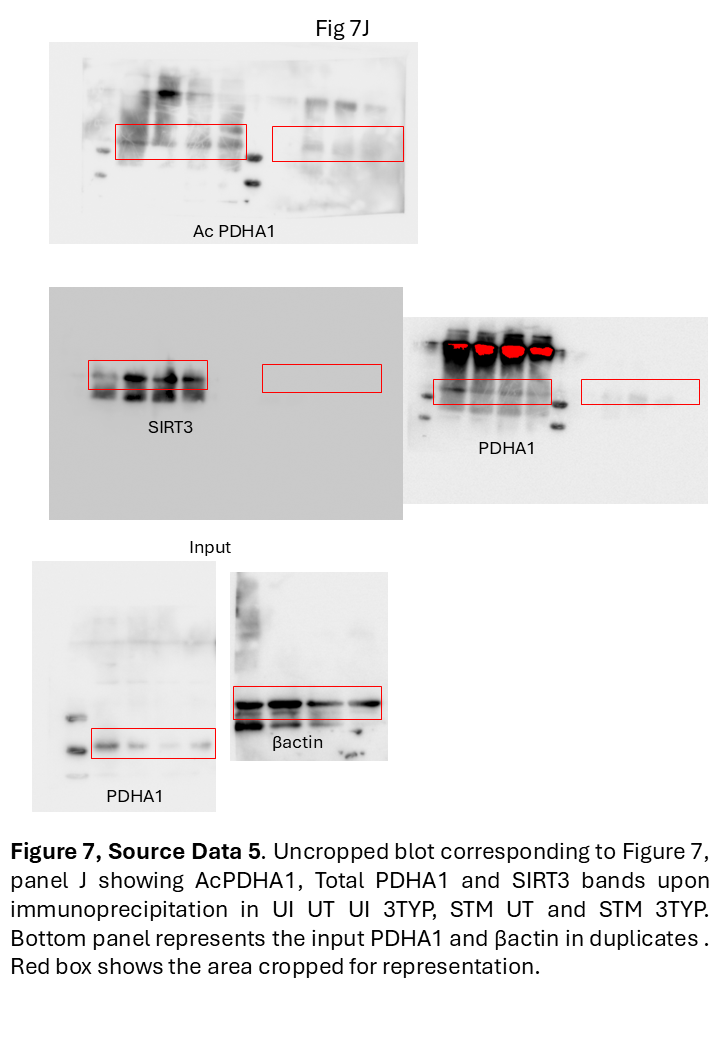

Supplement: Figure 7—source data 10. [file elife-93125-fig7-data10.zip › Figure 7-Source Data 5.tif]
